# Supplementary material for: Grain boundary engineering for efficient and durable electrocatalysis
Source: Nat Commun. 2024 Oct 2;15:8534. doi: 10.1038/s41467-024-52919-w (PMC11446910; doi:10.1038/s41467-024-52919-w)
Supplement: Supplementary file 1 — Supplementary Information [file 41467_2024_52919_MOESM1_ESM.pdf]

Grain Boundary Engineering for Efficient and Durable Electrocatalysis

Xin Geng[1*†], Miquel Vega-Paredes[1†], Zhenyu Wang[1*], Colin Ophus[2], Pengfei Lu[3], Yan Ma[1], Siyuan Zhang[1], Christina Scheu[1], Christian H. Liebscher[1], Baptiste Gault[1,4*]

[1] Max Planck Institute for Sustainable Materials; Düsseldorf 40237, Germany.

[2] National Center for Electron Microscopy, Molecular Foundry, Lawrence Berkeley National Laboratory; Berkeley 94720, USA.

[3] School of Energy and Power Engineering, Huazhong University of Science and Technology; Wuhan 430074, China.

[4] Department of Materials, Royal School of Mines, Imperial College London; London SW7 2AZ, UK.

*Corresponding author. Email: x.geng@mpie.de, z.wang@mpie.de, b.gault@mpie.de

†These authors contributed equally to this work.

Materials and Methods

1. Material preparation

Synthesis of OAm/OA-capped Au NPs. Initially, 25 mmol of 1-octadecene (ODE, 6.3 g, Sigma Aldrich) was subjected to heating at 130 °C for 30 min under N_2. Subsequently, a mixture comprising 2 mmol of oleic acid (OA, 0.56 g, Sigma Aldrich), 2 mmol of oleylamine (OAm, 0.53 g, Sigma Aldrich), 0.5 mmol of gold acetate (0.19 g, Sigma Aldrich), and 4 mmol of 1,2-hexadecanediol (1 g, Sigma Aldrich) was introduced. The reaction mixture was heated to 200°C and stirred for 2 h under N_2. Following this, the temperature was raised to 280°C and maintained for 1 h. Subsequently, the reaction was terminated by cooling the mixture to room temperature. The Au NPs were precipitated using ethanol and subsequently subjected to washing with hexane. The precipitation and washing steps were iteratively performed multiple times.

Preparation of citrate-capped Au NPs. A two-step methodology was employed to replace the capping agents on the surface of Au NPs from oleylamine (OAm)/oleic acid (OA) to citrate. The initial step involved the substitution of OAm/OA capping agents with diethanolamine (DEA, Sigma Aldrich). Specifically, 5 mL of OAm/OA-capped Au NPs were mixed with 5 mL of DEA (52 mmol, 5.5 g) and stirred for 24 h, followed by centrifugation for precipitation. Subsequently, 10 mL of DEA was added, and continuous stirring was maintained for 24 h, followed by another centrifugation step and dispersion in 5 mL of DI H_2O. Fourier-transform infrared spectroscopy (FTIR) analysis (**fig. S2**) conclusively confirmed the complete replacement of OAm/OA capping agents with DEA. The second step involved the replacement of DEA capping agents with citrate. Specifically, 5 mL of DEA-capped Au NPs were mixed with 1 mL of 10 mg/mL sodium citrate (Sigma Aldrich) and 1 mL of 10 mg/mL boric acid (Sigma Aldrich), followed by 24 h of stirring. Subsequent centrifugation and dispersion in 5 mL of DI H_2O were performed. FTIR results (**fig. S2**) provided unequivocal evidence for the full replacement of DEA with citrate capping agents.

Preparation of Au NAs. Initially, we employed a dialysis method to eliminate impurity ions from the citrate-capped Au NP solution. Specifically, the Au NP solution was placed inside dialysis tubing, which was immersed in a 1 L beaker containing DI water. Simultaneously, magnetic stirring was applied to expedite the diffusion of impurity ions away from the NP solution. The dialysis process was carried out continuously for three days, with DI water refreshed twice daily.

Subsequently, we introduced high-purity H_2 gas into a 10 mL solution of citrate-capped Au NPs, with a concentration of ~1.6 μM, and maintained this bubbling process for 10 h. To investigate the influence of H_2 gas flow rate on the assembly of Au NPs, we employed three distinct flow rates, denoted as low (30 sccm), medium (100 sccm), and high (300 sccm), resulting in the formation of Au NAs named L-Au NAs, M-Au NAs, and H-Au NAs, respectively. The completion of Au NAs assembly was indicated by a change in the color of the Au NP solution from dark brownish-red to transparent. After discontinuing the H_2 gas bubbling, the solution was allowed to stand for 10 h, during which the Au NAs sedimented at the bottom of the beaker. Subsequently, careful removal of the supernatant was performed using a pipette, followed by thorough rinsing of the Au NAs with DI water.

2. Characterization

Transmission Electron Microscopy Characterization. High-angle annular dark-field scanning transmission electron microscopy (HAADF-STEM) and high-resolution transmission electron microscopy (HR-TEM) were employed for characterization purposes. HAADF-STEM imaging was carried out using a Thermo Fisher Titan microscope with probe C_s correction at an accelerating voltage of 300 kV, while HR-TEM was performed utilizing an C_s image aberration-corrected Thermo Fischer Titan Themis 60-300 microscope, also operated at 300 kV. The HAADF-STEM and HR-TEM images were analyzed and post-processed utilizing the Gatan Microscopy Suite 3.0 (GMS 3.0) software package. To discern the crystallographic planes and zone axis, measurements of the interplanar spacing (d-spacing) and their respective angles were conducted through Fast-Fourier transformation (FFT) applied to the HAADF-STEM and HR-TEM images. In accordance with the coincidence site lattice theory, the types of grain boundaries (GB) existing between neighboring grains were identified. For better visualization, Hann-windowed FFT are provided in the text.
To map the strain of individual atoms, we employed STATSTEM[1, 2], a MATLAB-based program that allows for the fitting of two-dimensional Gaussians on the intensity maxima of HAADF-STEM images (i.e., the atomic columns) and posterior interatomic distance evaluation. After having calculated the interatomic distances, the strain of individual grains was analyzed by selecting the center of the corresponding grain as reference (unstrained) lattice. Afterwards, these strain maps of individual grains were combined for generating the complete strain map of the sample.

4D-STEM was used for performing grain orientation mapping and strain analysis of the nanoassemblies. The data was collected in the same C_s-probe corrected Thermo Fisher Titan microscope at 300 kV using the pixelated detector EMPAD. For the acquisition, a camera length of 940 mm and a probe convergence semiangle of 0.65 mrad were used. The detector pixel size was calibrated using a sample with (unstrained) Au NPs under the same conditions. The open source python library py4DSTEM was used for the preprocessing, visualization and orientation and strain mapping[3]. For the strain mapping, the relative strain is plotted. This is achieved by assuming that the average lattice parameter of the Au NAs is the same as that of an unstrained gold lattice. Using build in functions, the orientation maps were exported to ".ang" format, compatible with EDAX OIM Analysis software, where the grain misorientation angles were extracted for GB identification using coincidence site lattice theory. For that step, only the points with an image quality factor over 5.0 were considered to ensure a correct GB identification. Virtual dark field images were generated from the 4D-STEM datasets using a virtual annular detector centered around the bright field disk.

The GB surface density, defined as the length of the GB in the surface of the NAs (l_{GB}) divided by the surface area of the NAs (A_{NAs}) is assessed following a previously reported method:[4]

$$\text{GB surface density} = \frac{l_{GB}}{A_{NAs}} \qquad (1)$$

Assuming that the GBs assume a disk-like morphology, 'l_{GB}' is equivalent to '$2A_{GB}/r$', being A_{GB} the average area of a GB and 'r' the average GB projected length, estimated from the STEM images (100 GBs measured).

$$\text{GB surface density} = \frac{l_{GB}}{A_{NAs}} = \frac{2A_{GB}}{r \times A_{NAs}} \quad (2)$$

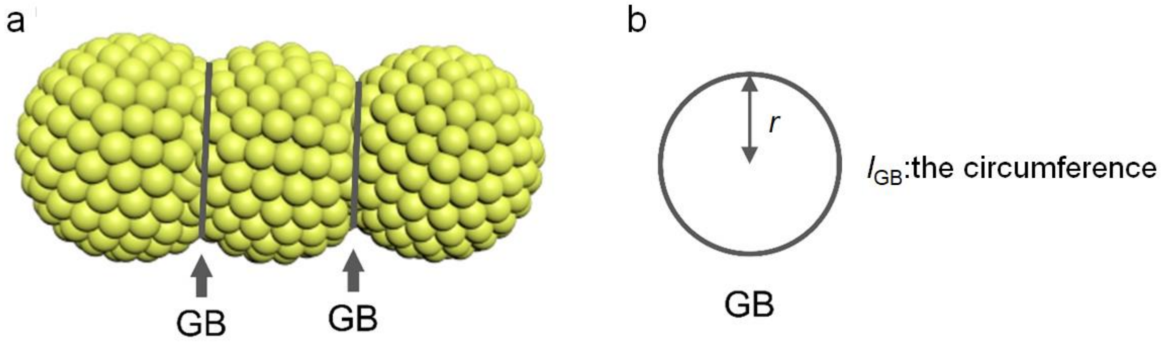

Figure S1. Geometric Definition of GB Surface Length and Area. (**a**) Schematic representation of GBs in NP assemblies. (**b**) The GB surface length (l_{GB}) can be defined as the circumference of a circular region with a radius denoted as '*r*', which is mathematically expressed as 2πr. Meanwhile, the GB surface area (A_{GB}) corresponds to the area enclosed by this circular boundary and can be quantified as πr^2.

Our investigations reveal that the crystallite size remains notably invariant both in pre- and post-NP assembly (**fig. S3-S4**). Consequently, the observed reduction in surface area within NAs in relation to their NP precursors is primarily attributed to the attachment of NPs. With that assumption, '$2A_{GB}$' can be expressed as the overall loss in surface area ($A_{NP}-A_{NAs}$), which can be experimentally determined via cyclic voltammetry to measure the electrochemically active surface area.

$$\text{GB surface density} = \frac{l_{GB}}{A_{NAs}} = \frac{2A_{GB}}{r \times A_{NAs}} = \frac{(A_{NP}-A_{NAs})}{r \times A_{NAs}} \quad (3)$$

Similarly, the surface area of the NAs (A_{NAs}) can also be experimentally measured via cyclic voltammetry.

X-ray Characterization. We investigated the crystal structures of Au NPs and Au NAs utilizing a Bruker Powder X-ray diffractometer, which was equipped with a Cu Kα radiation source. Subsequently, we employed an Al Kα X-ray Photoelectron Spectrometer (Thermo Scientific) to analyze the elemental composition and chemical states of Au NPs and Au NAs. To ensure precision in our X-ray photoelectron spectroscopy (XPS) spectra, we calibrated them with reference to the C1*s* peak at 284.8 eV. For the analysis and fitting of the XPS data, we utilized the Avantage software and employed a composite function (comprising 30% Lorentzian and 70% Gaussian components). To calculate the center of gravity (ε_d) for the valence band spectra for both Au NPs and Au NAs within the energy range of -10 eV to -1 eV, we utilized the following integral equation $\varepsilon_d = \frac{\int N(\varepsilon)\varepsilon d\varepsilon}{\int N(\varepsilon) d\varepsilon}$, where *N(ε)* represents the density of states and *ε* the energy level.

XAS, encompassing X-ray Absorption Near-Edge Spectroscopy (XANES) and Extended X-ray Absorption Fine Structure (EXAFS) measurements, were conducted at the BL14W1 beamline of the Shanghai Synchrotron Radiation Facility. The synchrotron storage ring operated at an energy of 3.5 GeV, while the linear electron accelerator operated at 150 MeV. Monochromatization of X-rays occurred through the utilization of a Si (311) double crystal

monochromator, operating within an energy range spanning 9,000–35,000 eV. The specimens were homogeneously mounted onto Kapton Tape for subsequent XAS assessments. XAFS spectra at the Au *L*3 edge were collected via transmission mode at a controlled temperature of 25°C. Rigorous data reliability measures were adopted, with all XAFS spectra being acquired within a defined beamtime and subjected to triplicate testing. Data preprocessing was facilitated through the employment of the Athena software, encompassing baseline subtraction prior to post-edge baseline normalization to yield the spectroscopic data. Furthermore, the EXAFS analysis involved Fourier transformation of *k*3-weighted EXAFS oscillations to assess the contribution of each shell to the peaks in the Fourier transform, followed by fitting using the Artemis software.

Atom Probe Tomography (APT) Characterization. Initially, the incorporation of Au NPs and Au NAs into Ni matrix was achieved through the application of a co-electrodeposition technique. Specifically, a copper foil (0.2 cm^2) was employed as working electrodes (pre-etched by 0.5 M H_2SO_4), while a Pt mesh (2 cm^2) served as the counter electrode. An electrolyte solution consisting of 1.5 g $NiSO_4 \cdot 6H_2O$ and 0.225 g citric acid dissolved in 5 mL of DI water was prepared. Subsequently, a mixture of 10 mg of Au NPs and Au NAs was sonicated with the electrolyte for 1 h, and the resulting solution was poured into an electrodeposition cell. An established constant current of -19 mA was applied to the working electrode for 500 s, resulting in the deposition of an Au/Ni thin film, with Au NPs and Au NAs encapsulated within the Ni matrix. Following this, needle-shaped APT samples were prepared using a Ga focused ion beam (FEI 600 DualBeam) in accordance with standard APT sample fabrication procedures. The embedding of Au NPs or Au NAs within the Ni matrix led to surface protrusions on the Ni film, which were meticulously sectioned and transferred onto silicon coupons as lamellas. The lamella containing Au NPs or Au NAs encapsulated within the Ni matrix was verified through cross-sectional analysis of the protrusion, followed by precise sharpening to achieve a needle-shaped APT specimen. Finally, the needle-shaped APT sample was loaded into the LEAP 5076XS instrument (Cameca) for APT experiments. All APT experiments were conducted in pulse laser mode, with experimental parameters set at a temperature of 50 K, a detection rate of 1%, a laser energy of 60 pJ, and a laser pulse frequency of 125 kHz. Data obtained were analyzed and reconstructed using standard voltage reconstruction protocols with the assistance of the commercially available IVAS 3.8.4 software. For APT, the broadening of segregation profiles at GBs is a frequently observed phenomenon attributed to the distinct field evaporation behavior at these interfaces. In order to discern the segregation behavior independently of the broadening effects, we employ the Gibbsian interfacial excess (Γ) as an integrated metric. The computation of Γ_B is based on the following equation: $\Gamma_B = t \times \Delta\rho_B = t \times (\frac{N_B^{GB}}{V_{GB}} - \frac{N_B^{NP}}{V_{NP}})$, wherein t represents the width of the GB region, $\Delta\rho_B$ signifies the difference in atomic density of the B element between the NP building blocks and the GB region, N_B^{NP} and N_B^{GB} denote the number of atoms of the B element in the respective unit cells of the NP building block and the GB region, while V_{NP} and V_{GB} represent the unit cell volumes of the NP building block and the GB region.

3. Theoretical calculation

Density functional theory (DFT) calculations. We conducted DFT calculations employing the Vienna Ab initio Simulation Package (VASP) in conjunction with the Atomic Simulation Environment (ASE) [5-11]. To account for spin polarization effects, appropriate adjustments were applied. The exchange correlation energy was assessed utilizing the generalized gradient approximation (GGA) method[12], specifically employing the revised Perdew-Burke-Ernzerhof (RPBE) functional[13]. To accurately describe the behavior of core electrons, we employed the projector-augmented wave (PAW) method[14, 15]. To ensure precision, lattice constants within our calculations were systematically optimized according to their respective crystal systems. The (111) surfaces of various metals and alloys (Au, Hg, Pt, Pd, Ir, Rh, PdAu, PdHg) were modeled using six-layer slabs, with the bottom three layers fixed and the upper three layers and adsorbates allowed to relax. To investigate the effect of coordination number on two-electron ORR activity, slab models of the Au (111) surface with different coordination numbers were used for OOH binding energy calculations (**fig. S25**). Additionally, to examine the impact of GB type on two-electron ORR activity, slab models of Σ3, Σ9, and Σ27 GB were employed for OOH binding energy calculations (**fig. S26**). For sampling the Brillouin zones, we utilized a 4×4×1 Monkhorst-Pack k-points grid[16]. To mitigate interlayer interactions, a minimum separation of at least 16 Å of vacuum was enforced between successive slabs in all computational simulations. Furthermore, an energy cutoff of 500 eV was imposed, and all structures were subjected to relaxation in all spatial dimensions until residual forces reached a level below 0.05 eV/Å, ensuring the attainment of thermodynamically stable configurations.

The computation of adsorption energies was carried out with reference to H_2O (l) and H_2 (g):

$$\Delta E_{*OOH} = E_{*OOH} - E_{*} - E_{H2O} + 3/2E_{H2} \qquad (4)$$

Zero-point energies and entropy terms have been encompassed within the framework of our energetic computations:

$$\Delta G^0 = \Delta E_{DET} + \Delta E_{ZPE} - T\Delta S \qquad (5)$$

Under an arbitrary potential, relative to RHE, the electron's chemical potential undergoes a perturbation of -eU. Consequently, the reaction's free energy is expressed as:

$$\Delta G = \Delta G_0 + eU \qquad (6)$$

The determination of the limiting potential (U_L) is formulated as follows:[17]

$$U_L = \min(-\Delta G_{*OOH} + 4.92, \Delta G_{*OOH} - 3.52) \qquad (7)$$

The two constituent terms within the aforementioned equation correspond to the limiting potentials associated with the individual elementary steps comprising the two-electron oxygen reduction pathway. It is worth noting that the lower of these limiting potentials, characterizing the complete catalytic reaction, establishes the overarching limiting potential governing the overall reaction kinetics.

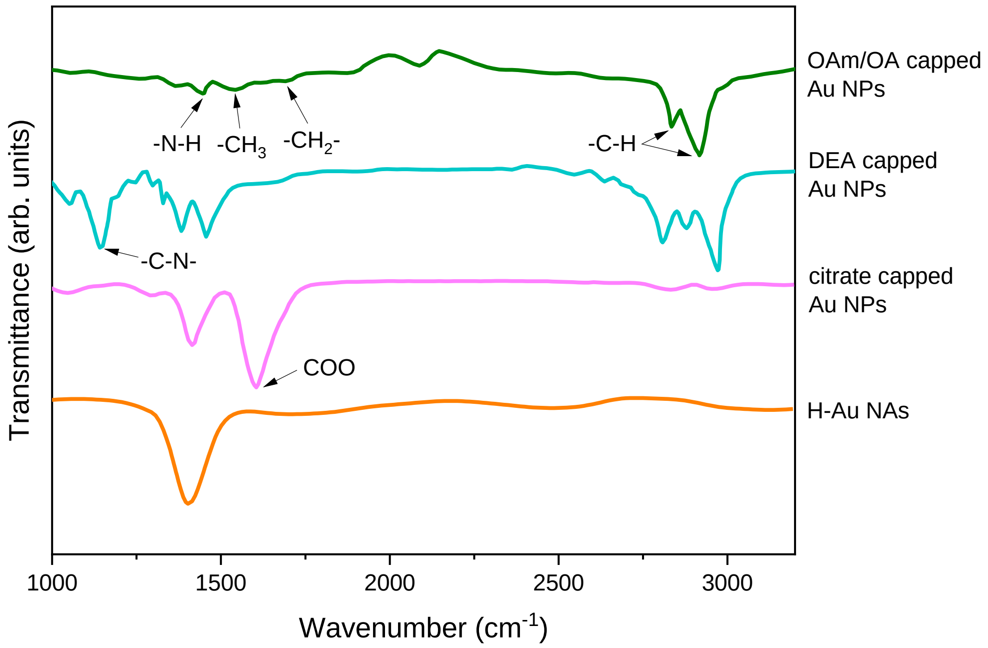

Figure S2. FTIR Spectra of Au NPs and NAs. FTIR spectra of oleylamine (OAm), diethanolamine (DEA), citrate-capped Au NPs, and Au NAs.

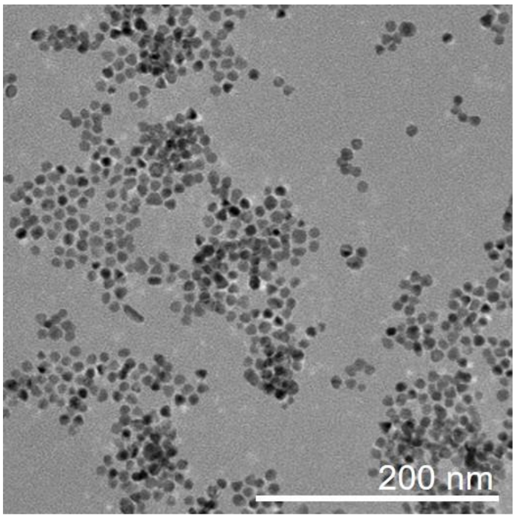

Figure S3. TEM Image of Au NPs. TEM image showing the morphology and dispersion of Au NPs at low magnification.

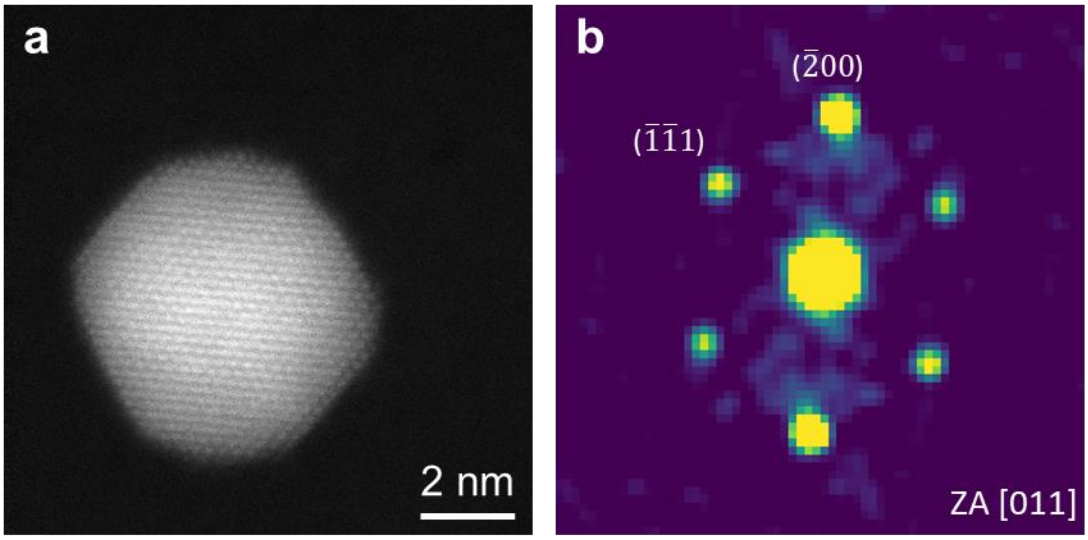

Figure S4. High-Resolution HAADF-STEM Image of Au NP Along the [011] Zone Axis. (**a**) High-resolution HAADF-STEM image of an Au NP oriented along the [011] zone axis. (**b**) Corresponding FFT pattern.

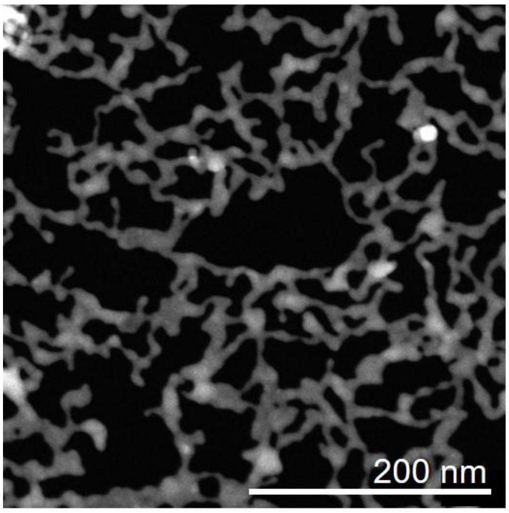

Figure S5. HAADF-STEM Image of H-Au NAs. Low-magnification HAADF-STEM image of H-Au NAs.

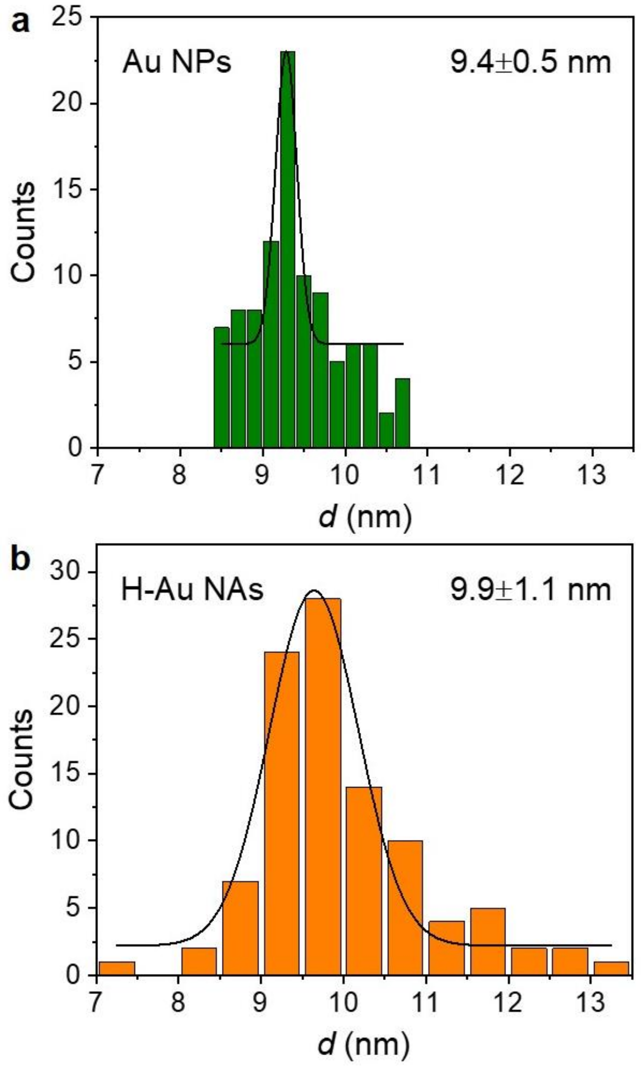

Figure S6. Size Distribution of Au NPs and H-Au NAs. The size distribution was determined by analyzing 100 NPs in (**a**) Au NPs and 100 building blocks in (**b**) H-Au NAs, as observed in STEM images.

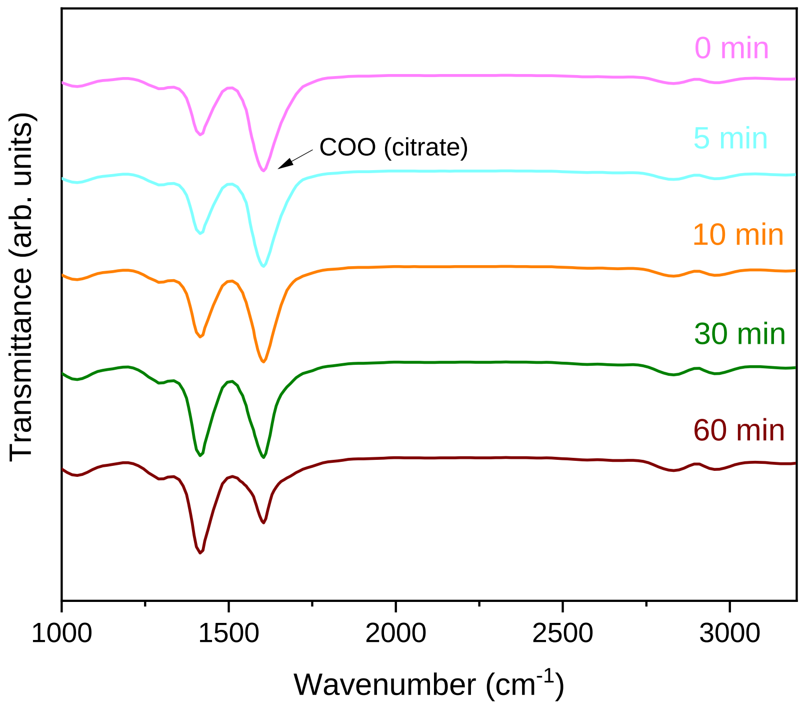

Figure S7. Variation of FTIR Spectra Following Ammonia Addition to Citrate-Capped Au NP Solution. Variation of FTIR spectra for citrate-capped Au NP solution after adding ammonia solution (pH=10), which show that the amount of citrate capping agents on the surface Au NPs reduces progressively.

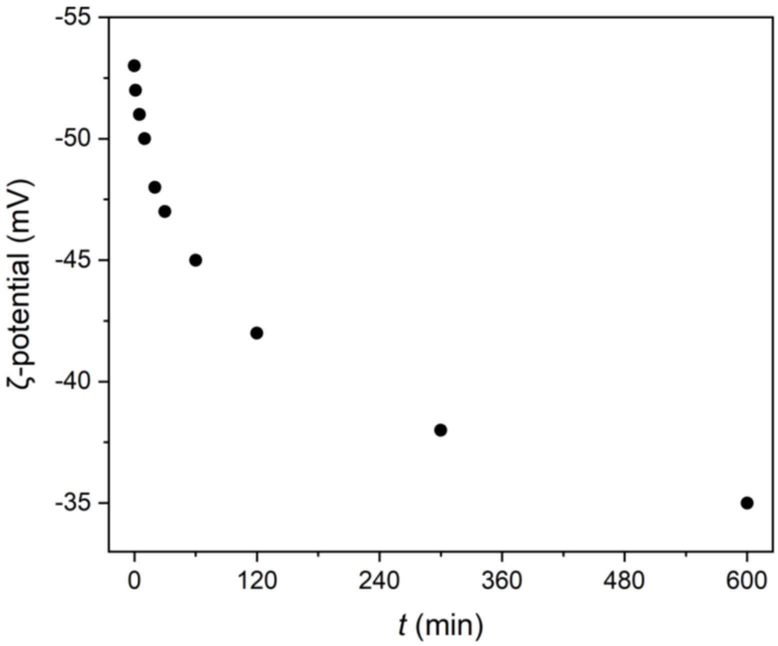

Figure S8. Variation of ζ-Potential in Citrate-Capped Au NP Solution. Variation of the ζ-potential measured for citrate-capped Au NP solution versus the time after adding ammonia solution (pH=10).

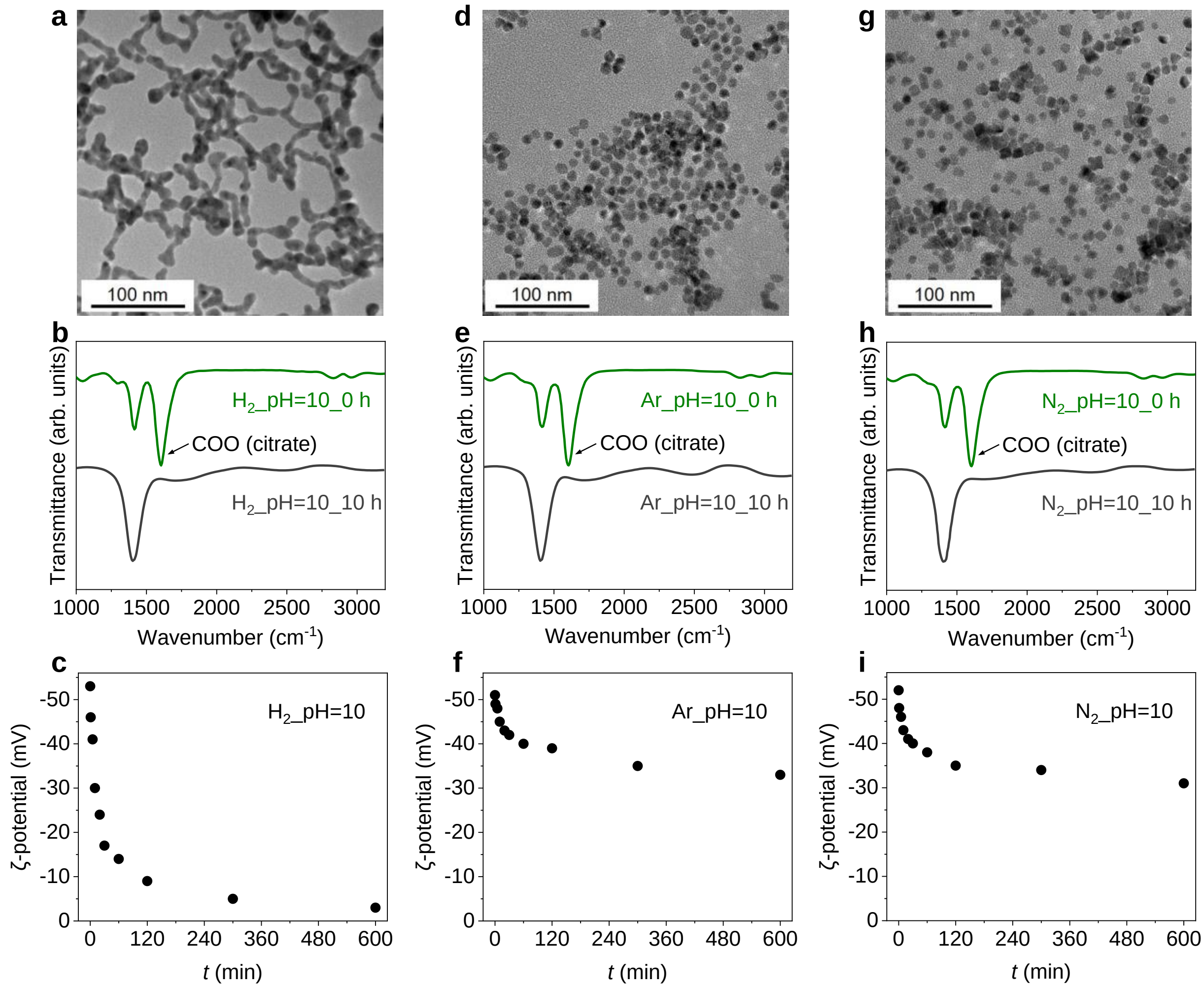

Figure S9. Characterization of Citrate-Capped Au NPs Under Various Gas Purging Conditions. (**a**) TEM image of the NPs obtained by purging Ar gas into a citrate-capped Au NP solution at pH 10 for 10 h. (**b**) FTIR spectrum of the Au NP solution at pH 10 subjected to Ar gas purging for 0 h and 10 h, respectively. (**c**) Time-dependent variation of the ζ-potential for citrate-capped Au NP solution at pH 10 during Ar gas purging. TEM image (**d**), FTIR spectrum (**e**) and time-dependent variation of ζ-potential (**f**) for citrate-capped Au NP solution at pH 10 during N_2 gas purging. TEM image (**g**), FTIR spectrum (**h**) and time-dependent variation of ζ-potential (**i**) for citrate-capped Au NP solution at pH 10 during H_2 gas purging.

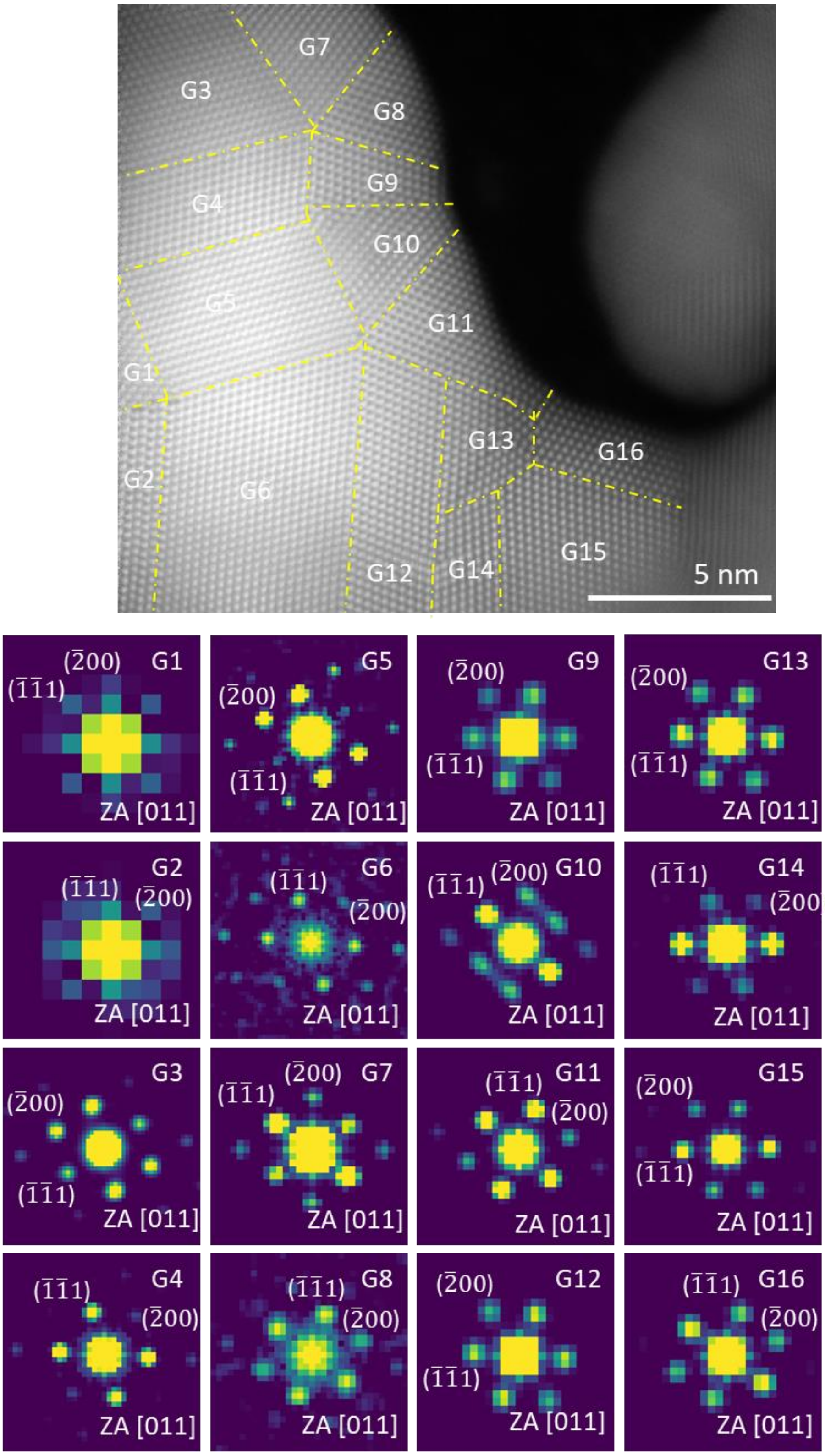

Figure S10. FFT Analysis of H-Au NAs. Fast Fourier Transform (FFT) analysis of the NA displayed in **Figure 1b**. From the FFT diffractograms (bottom rows of **Figure S10**) of individual NP building blocks in the HAADF-STEM image (top row of **Figure S10**), the zone axis ([011] for all NP building blocks) and missorientation can be determined. This allows for the identification of the GB types in H-Au NAs using coincidence site lattice theory.

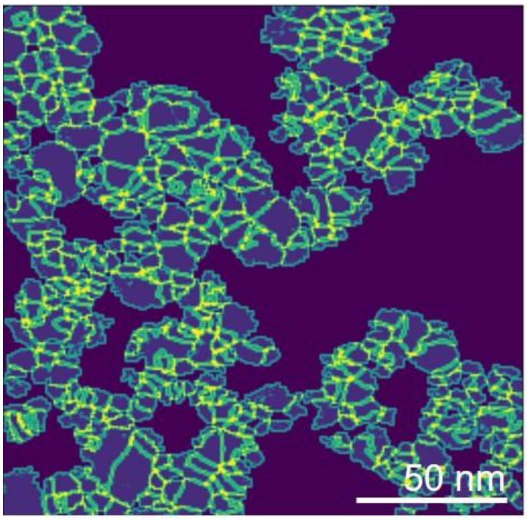

Figure S11. 4D-STEM Map Illustrating GBs. The 4D-STEM map highlighting the boundaries between the grains is given.

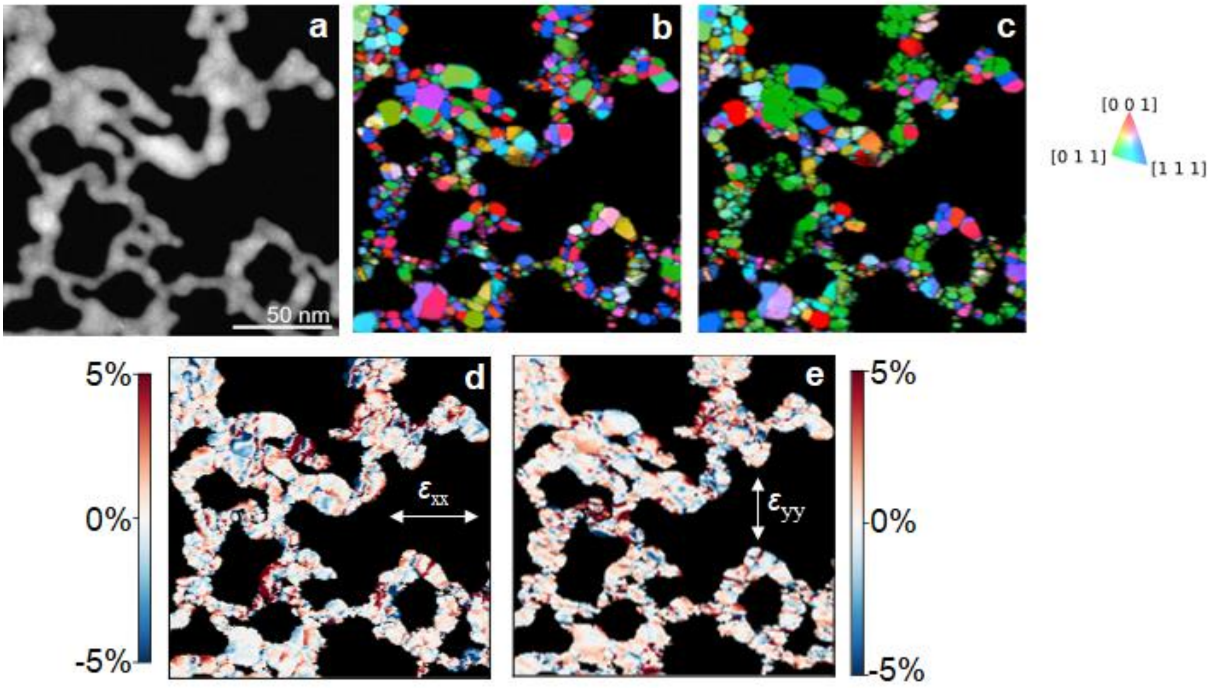

Figure S12. GB and Strain Mapping of region of interest 1 in H-Au NAs using 4D-STEM. (**a**) HAADF-STEM image, (**b-c**) grain orientation maps, and corresponding strain maps showing relative strain in the (**d**) x (ε_{xx}) and (**e**) y (ε_{yy}) directions. The data were obtained from 4D-STEM analysis of region of interest 1 in H-Au NAs.

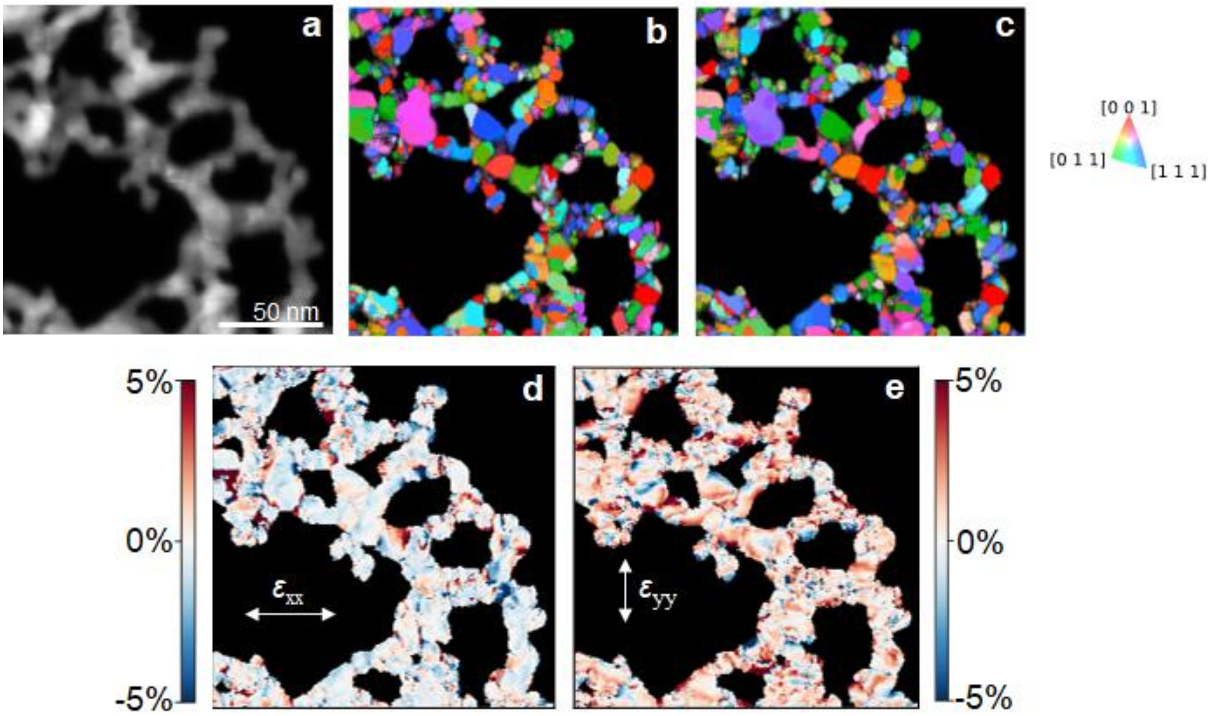

Figure S13. GB and Strain Mapping of region of interest 2 in H-Au NAs using 4D-STEM. (**a**) HAADF-STEM image, (**b-c**) grain orientation maps, and (**d-e**) relative strain maps along the x (ε_{xx}) and y (ε_{yy}) directions, respectively, obtained from 4D-STEM datasets for region of interest 2 in H-Au NAs.

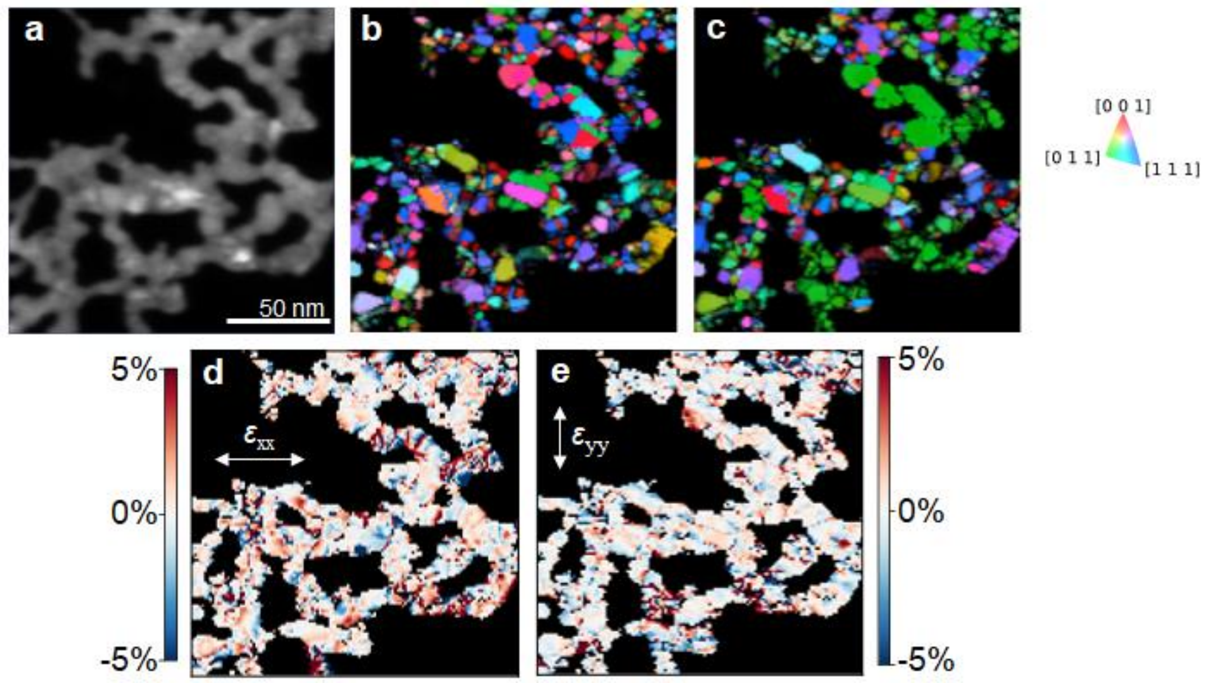

Figure S14. GB and Strain Mapping of region of interest 3 in H-Au NAs using 4D-STEM. (**a**) HAADF-STEM image, (**b-c**) grain orientation maps, and (**d-e**) relative strain maps along the x (ε_{xx}) and y (ε_{yy}) directions, respectively. These data are derived from 4D-STEM datasets for region of interest 3 in H-Au NAs.

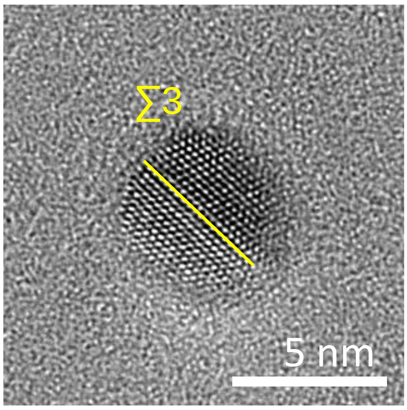

Figure S15. High-Resolution TEM Image of Individual Au NP. High resolution TEM image of an individual Au NP, showing that a a small number of Au NPs possess low energy ∑3 GBs before assembling.

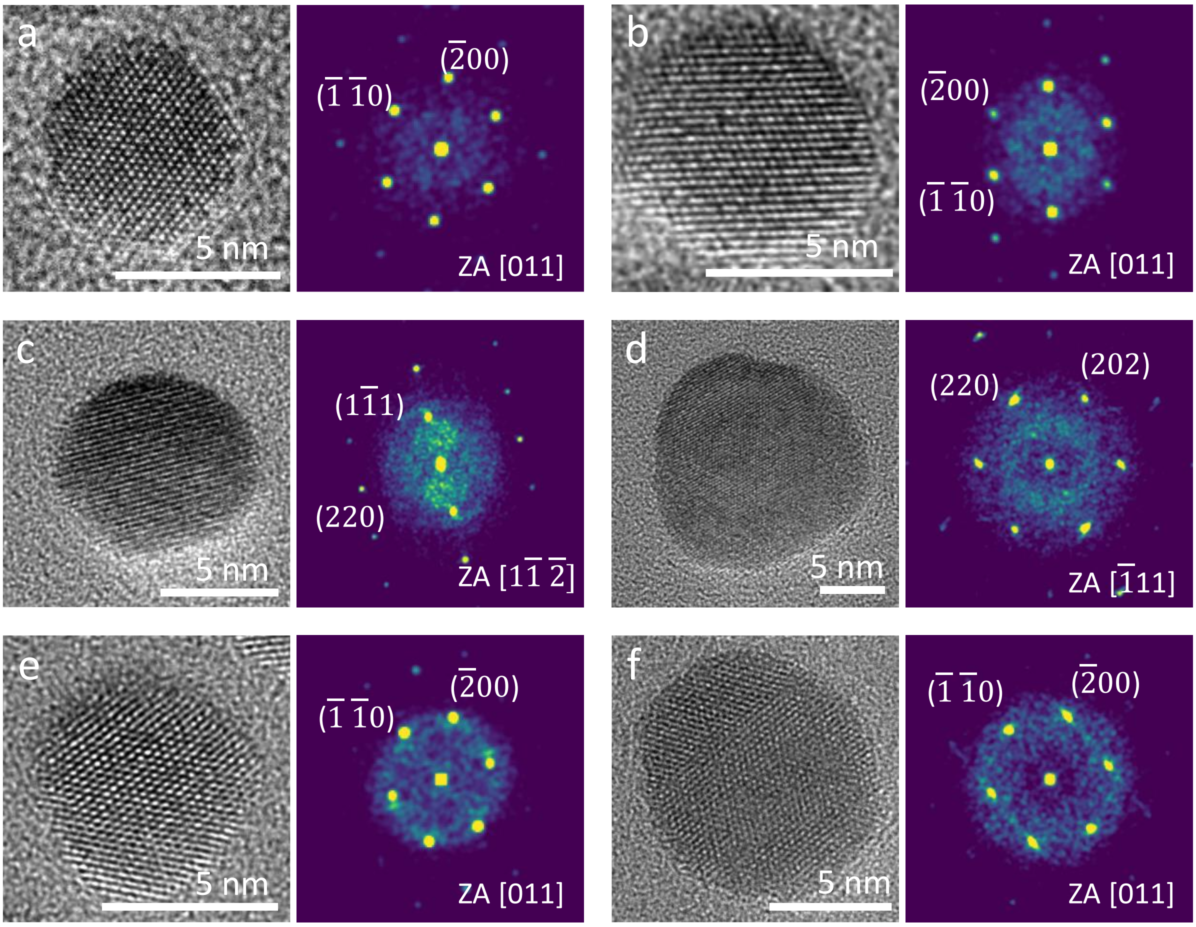

Figure S16. High-Resolution TEM and FFT Analysis of Au NPs Prior to Assembly. (a-f) High-resolution TEM images of Au NPs of interest 1 through 6, with corresponding indexed fast Fourier transform patterns. The images reveal that most Au NPs are defect-free before the assembly process.

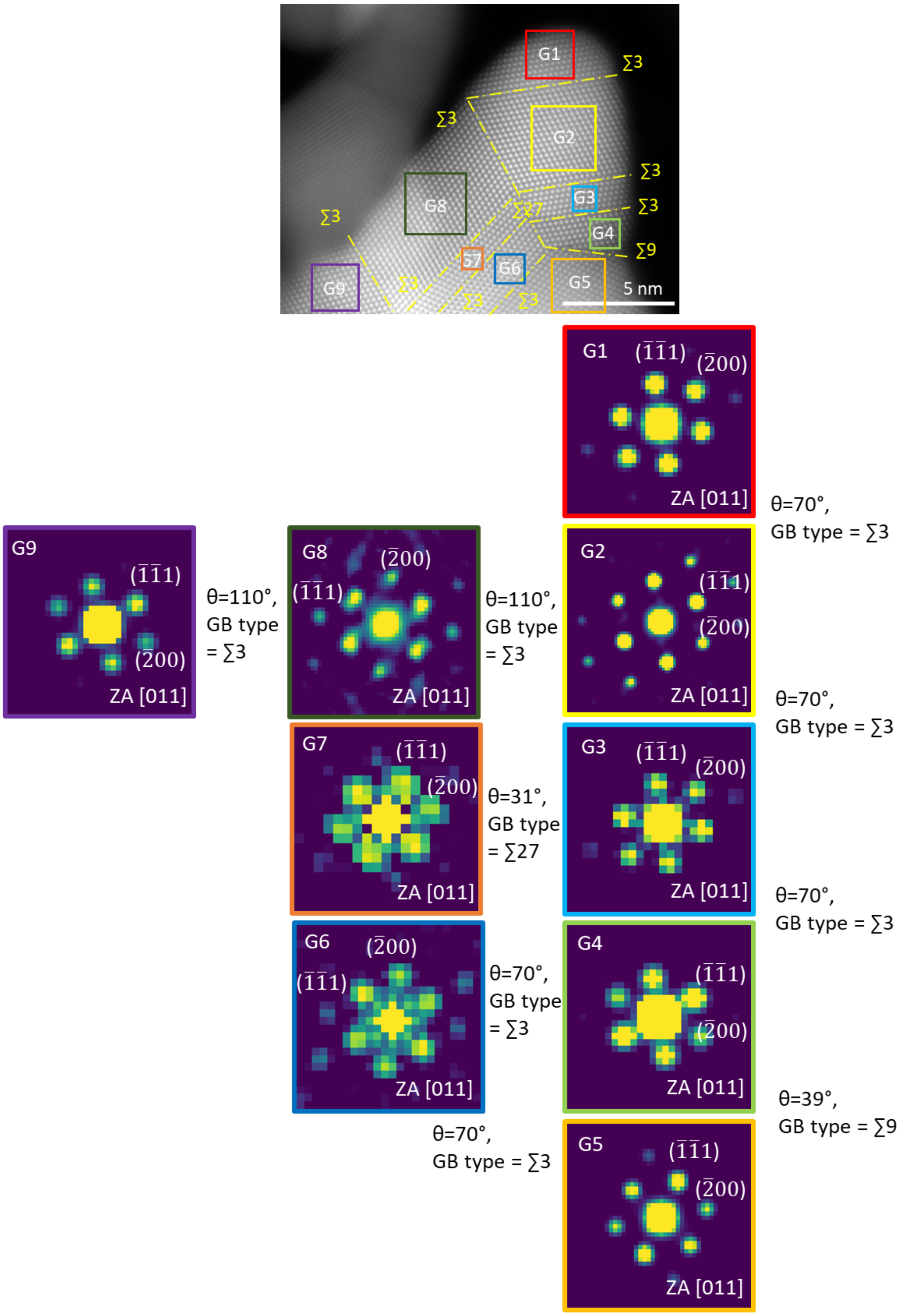

Figure S17. Identification of GB Types Using Coincidence Site Lattice Theory. Determination of GB types based on the coincidence site lattice theory of NA in **Figure 2a** of main text. Initially, FFT diffractograms (bottom row of **Figure S17**) were obtained for individual NP building blocks in HAADF-STEM image (top row of **Figure S17**) to determine the zone axis (ZA). Subsequently, the misorientation between adjacent NP building blocks on the same crystal plane was measured, allowing for the identification of the GB types in H-Au NAs.

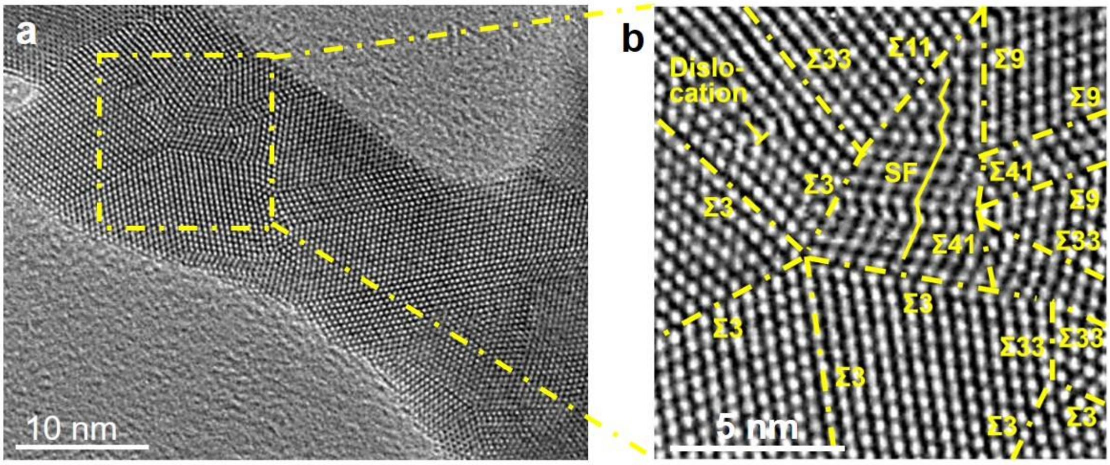

Figure S18. HR-TEM Images of H-Au NAs. (**a**) Low- and (**b**) high-magnification HR-TEM images of H-Au NAs. The different defects present in the crystal lattice are indicated.

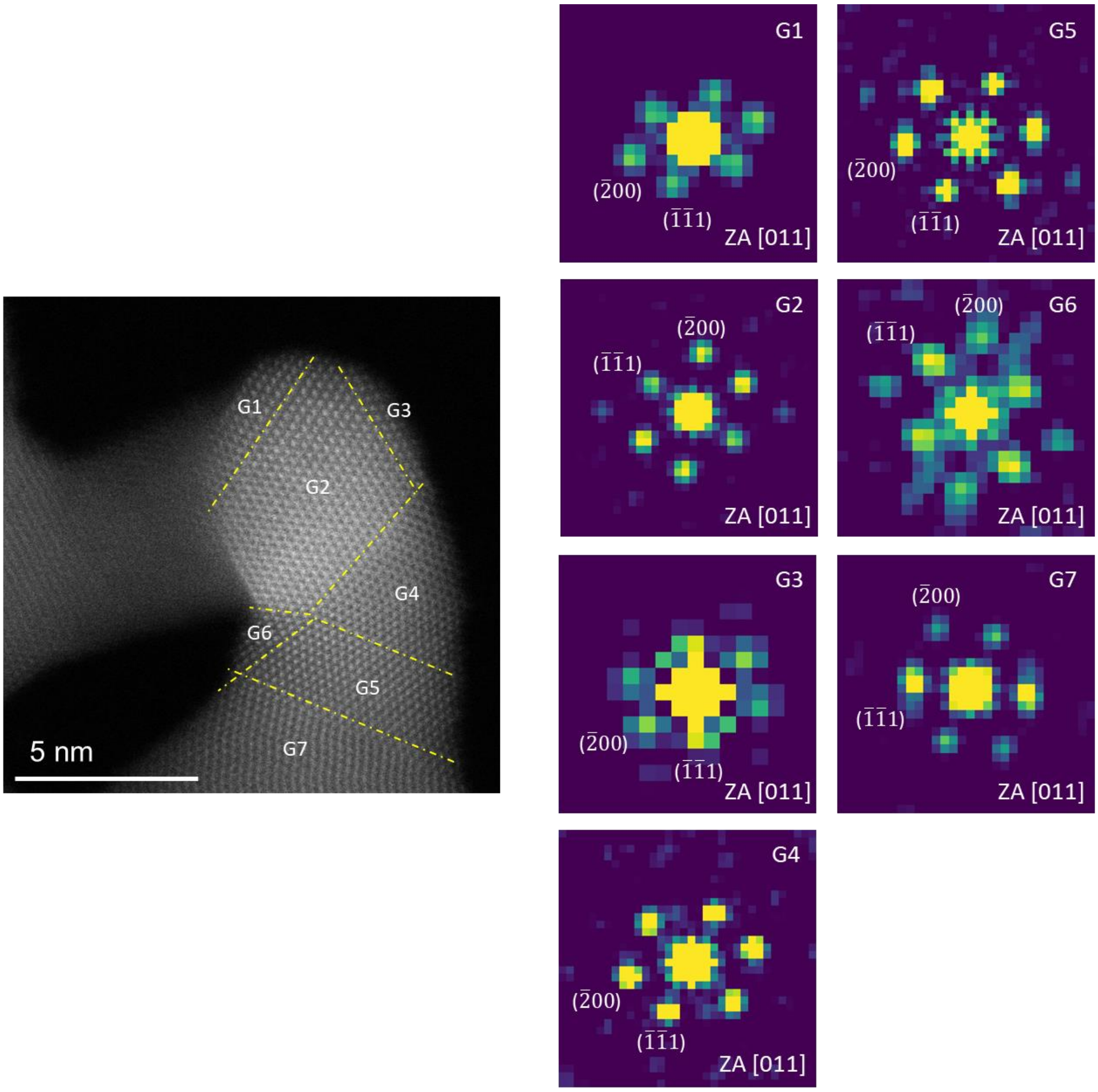

Figure S19. Identification of GB Types in L-Au NAs. HAADF STEM image and corresponding FFT analysis for the identification of GB types in L-Au NAs of **Figure 3d** (main text) based on the coincidence site lattice theory.

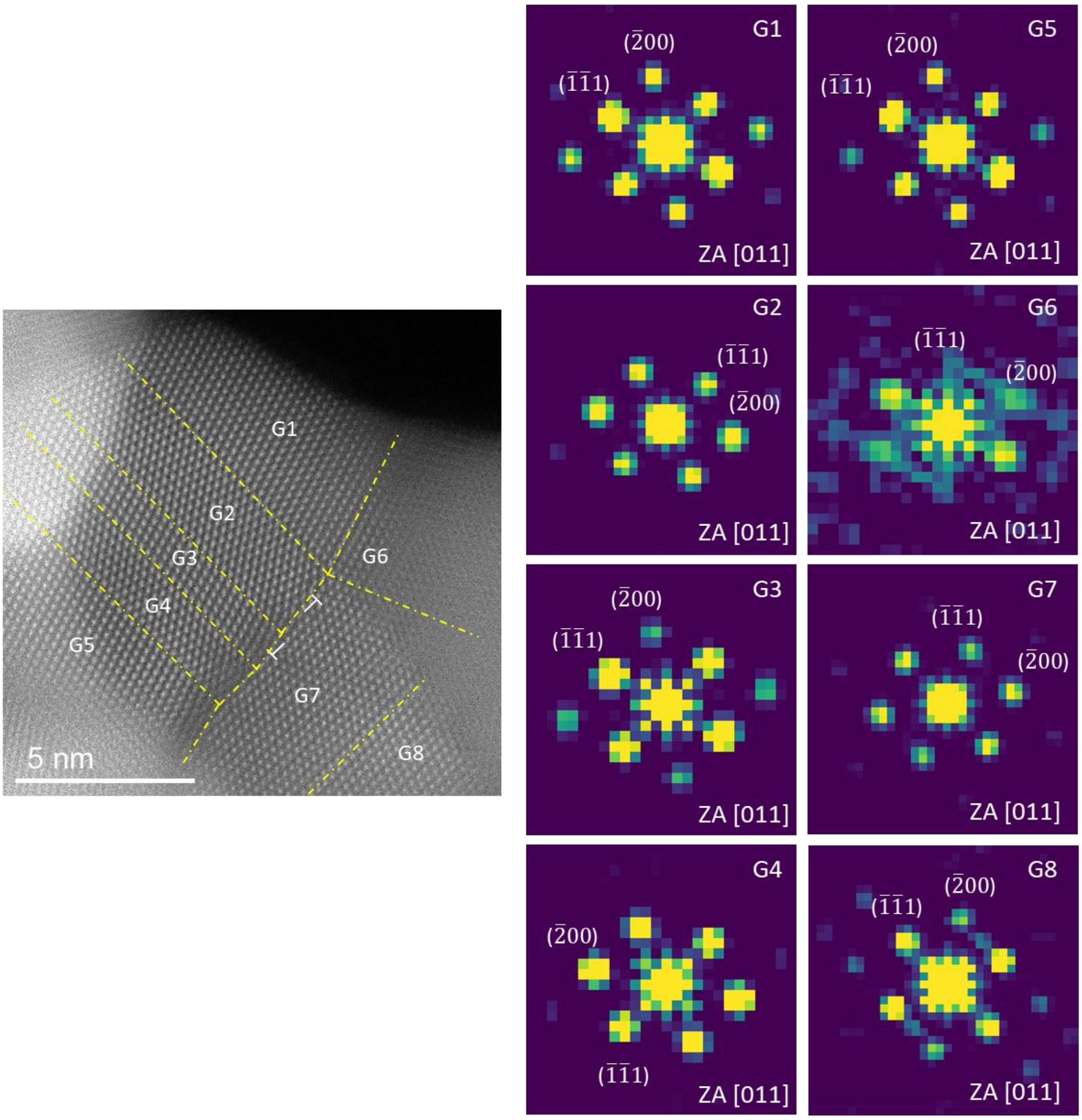

Figure S20. Identification of GB Types in M-Au NAs. HAADF-STEM image and corresponding FFT analysis of M-Au NAs (**Figure 3e**, main text) for GB identification.

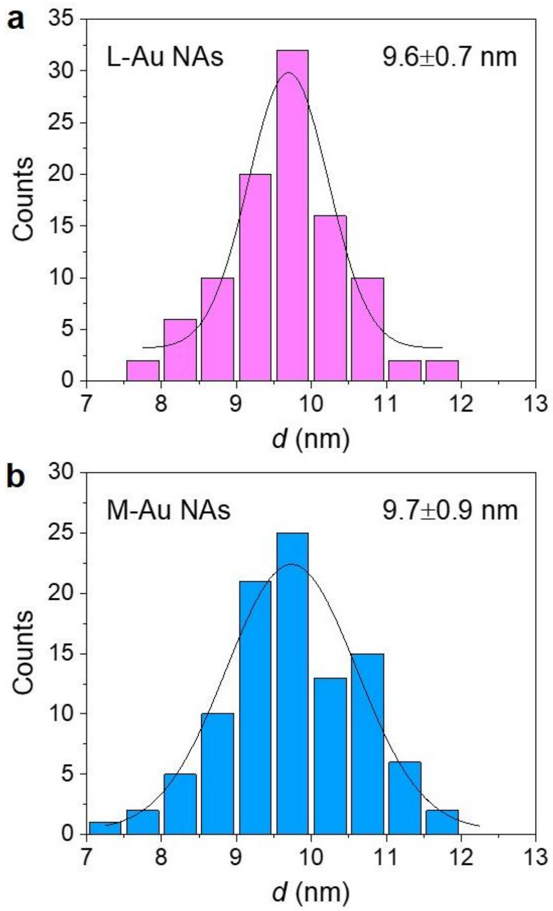

Figure S21. Size Distribution of L-Au NAs and M-Au NAs. The size distribution was obtained by analyzing 100 NPs or crystallites in (**a**) L-Au NAs and (**b**) M-Au NAs derived from STEM images.

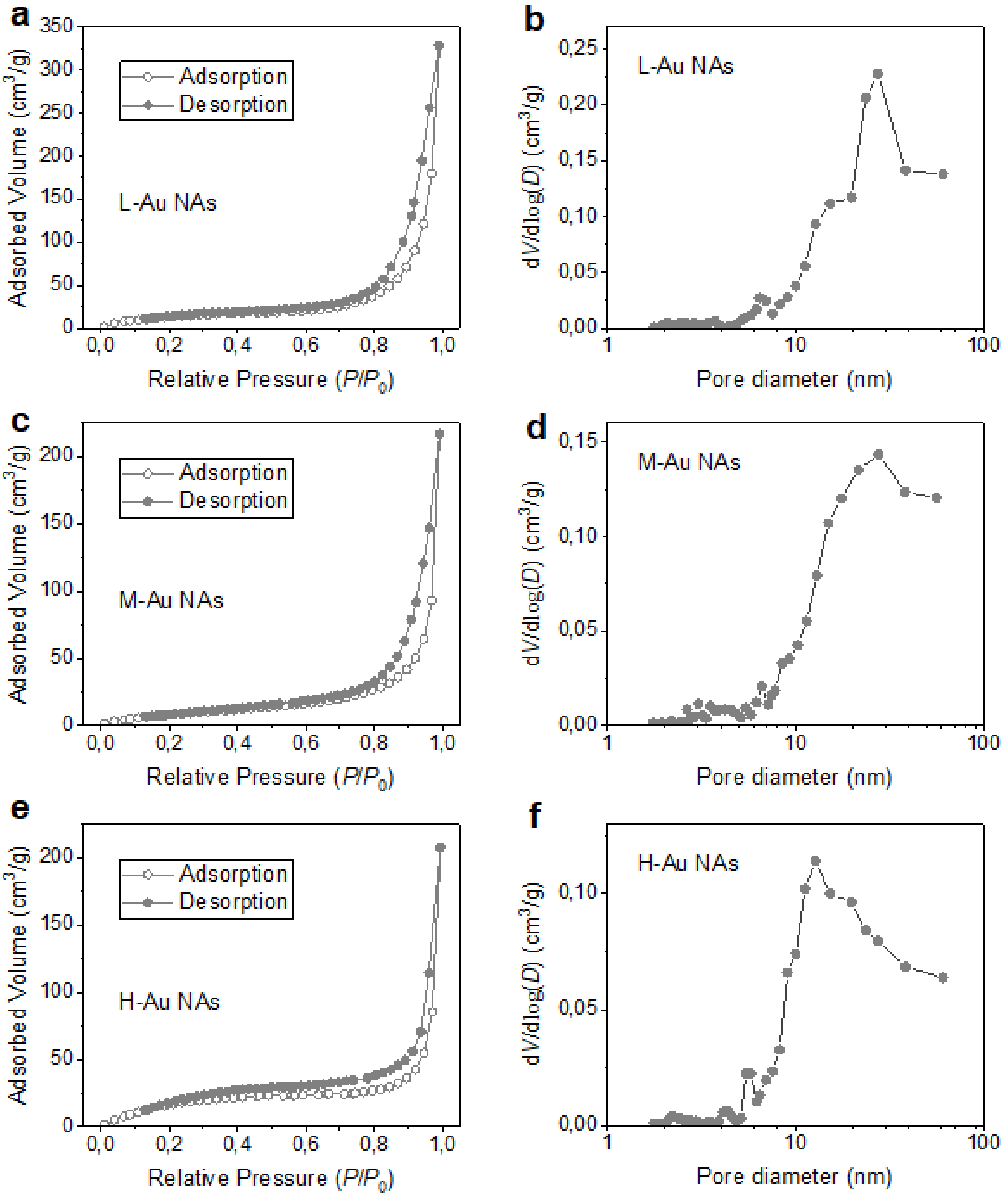

Figure S22. Nitrogen Adsorption–Desorption Isotherms and Pore Size Distribution of Au NAs. Nitrogen adsorption–desorption isotherms of L-Au NAs (**a**), M-Au NAs (**c**) and H-Au NAs (**e**). Barrett–Joyner–Halenda pore size distribution plots of L-Au NAs (**b**), M-Au NAs (**d**) and H-Au NAs (**f**).

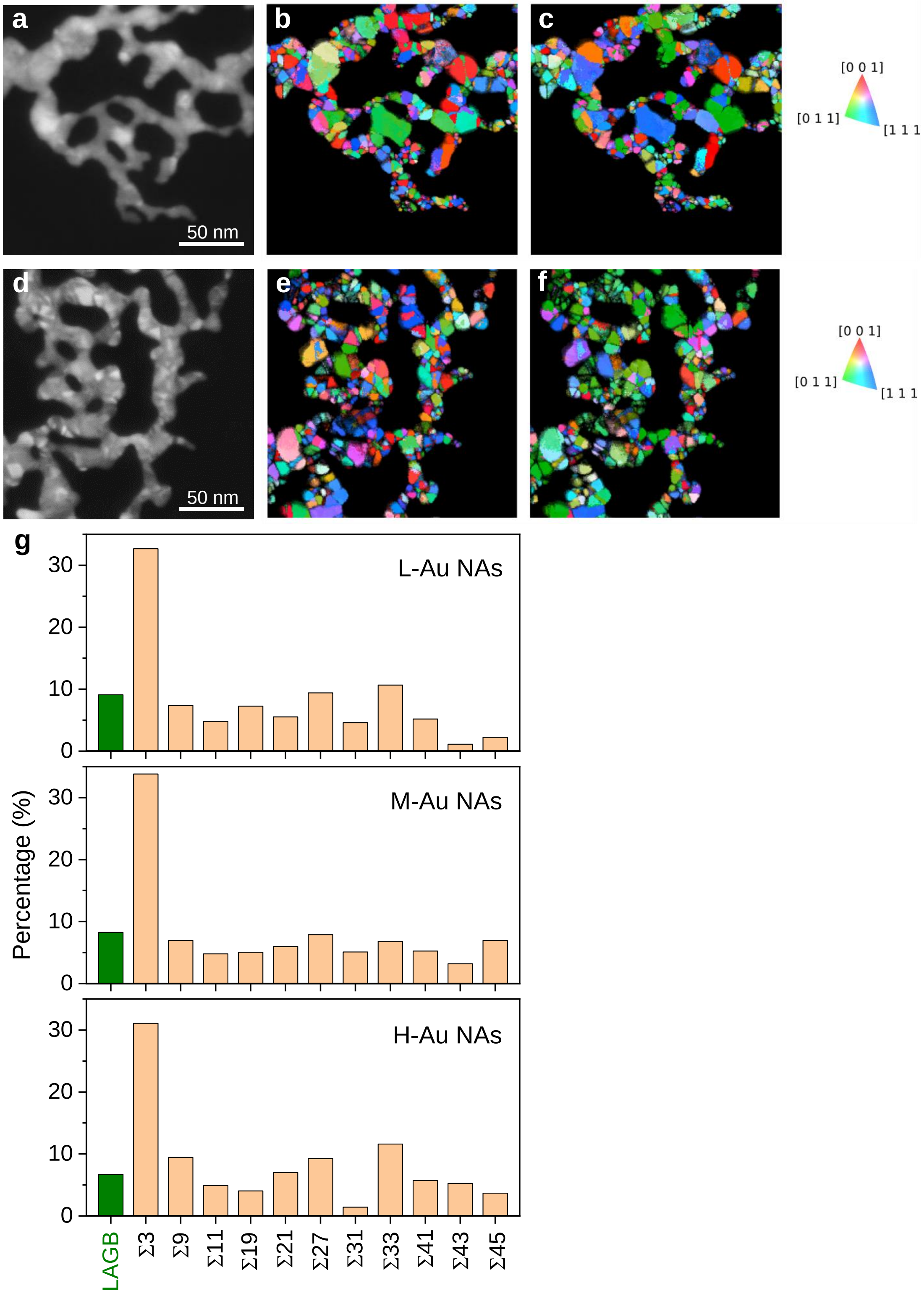

Figure S23. GB Type Analysis of L-Au and M-Au NAs using 4D-STEM. (**a**) HAADF-STEM image, (**b**) in-plane and (**c**) out-of-plane orientation maps from the corresponding 4D-STEM dataset for L-Au NAs. (**d**) HAADF-STEM image and grain orientation maps (**e**: in-plane, **f**: out-of-plane) from the corresponding 4D-STEM dataset for M-Au NAs. (**g**) Histogram plots of the GB types derived from over 100 GBs in the 4D-STEM data.

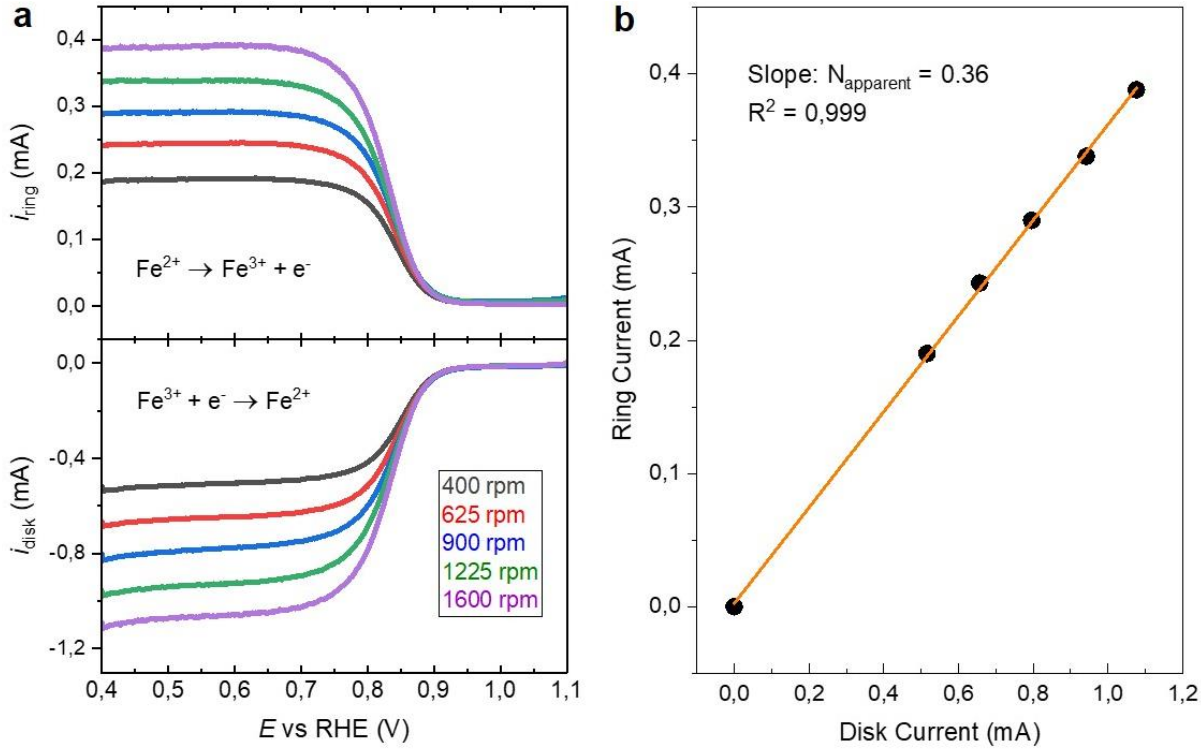

Figure S24. Calibration of RRDE Collection Efficiency. RRDE collection efficiency calibration. (**a**) Linear sweep voltammetry curves recorded on a bare glassy carbon rotation disk electrode (Φ = 5.6 mm) with a Pt ring (Φ = 15.0 mm) in 0.1 M $HClO_4$ supporting electrolyte with 10 mM $K_3Fe(CN)_6$. Sweep rates: 10 mV s^{-1}, E_{ring} = 1.2 V vs. RHE. (**b**) Linear fitting of the diffusion limited current densities recorded on ring and disk electrodes at different rotation speed. The experimental determined apparent collection efficiency (N) is 36%, close to the theoretical vale of 40%.

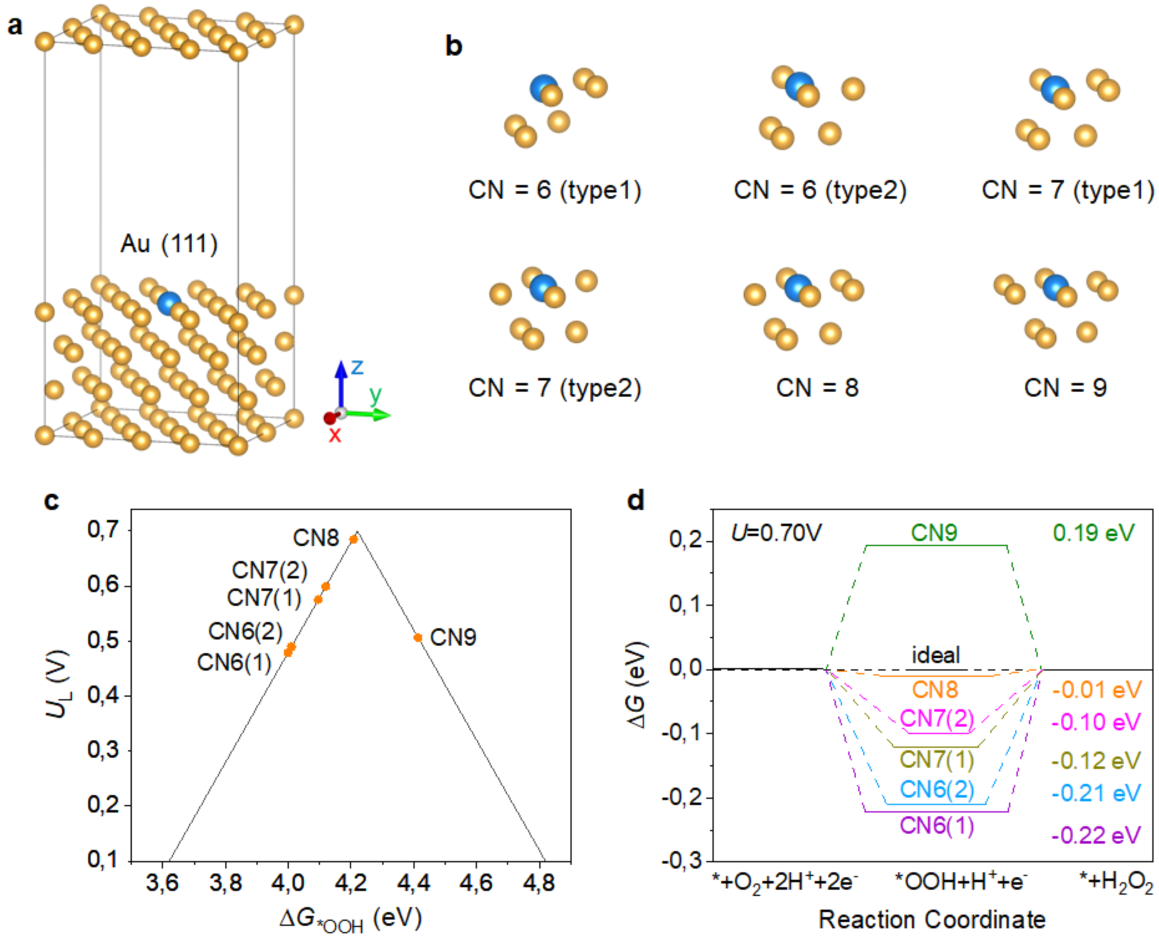

Figure S25. Modeling and Activity Analysis of Au (111) Surface for OOH Binding. (**a**) Slab model of the Au (111) surface, highlighting the surface atom used for OOH binding energy calculations in blue. (**b**) Surface atom (blue) used for OOH binding energy calculations with different coordination numbers (CNs) on the Au (111) surface, showing only the nearest neighbor atoms. (**c**) Calculated ORR activity volcano relationship between the limiting potential (U_L) and the free energy of *OOH (ΔG_{*OOH}) for the two-electron pathway to H_2O_2. (**d**) Calculated reaction coordinate diagrams for Au with different CNs.

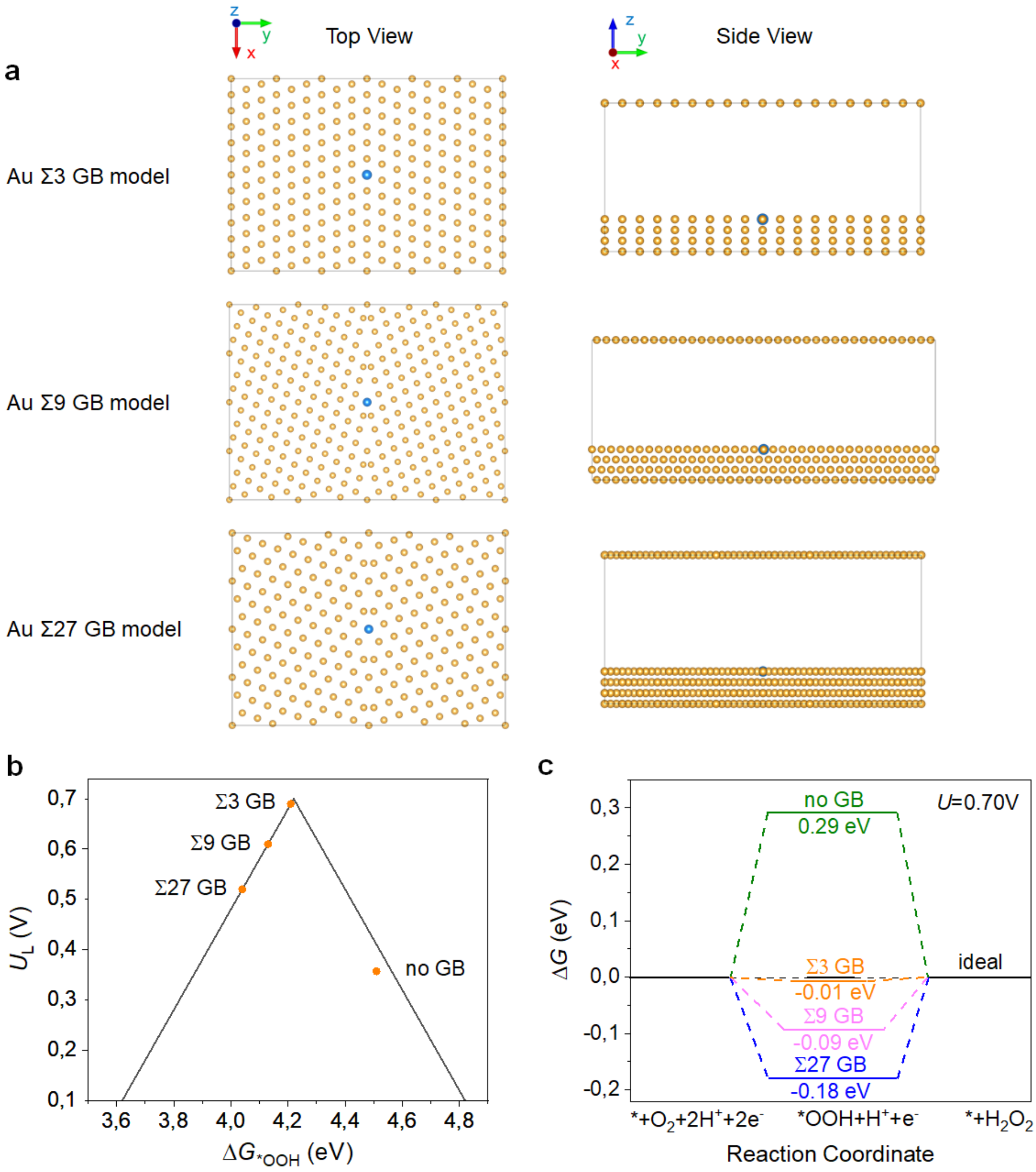

Figure S26. Models and Activity Analysis of OOH Binding on Au GBs. (**a**) Top and side views of the models used to calculate the OOH binding energy on Au Σ3 GB, Σ9 GB, and Σ27 GB. The surface atom used in these calculations is highlighted in blue. (**b**) Calculated ORR activity volcano plot showing the relationship between the limiting potential (U_L) and the free energy of *OOH (ΔG_{*OOH}) for the two-electron pathway to H_2O_2 on Au Σ3 GB, Σ9 GB, and Σ27 GB. (**c**) Calculated reaction coordinate diagrams for Au Σ3 GB, Σ9 GB, and Σ27 GB.

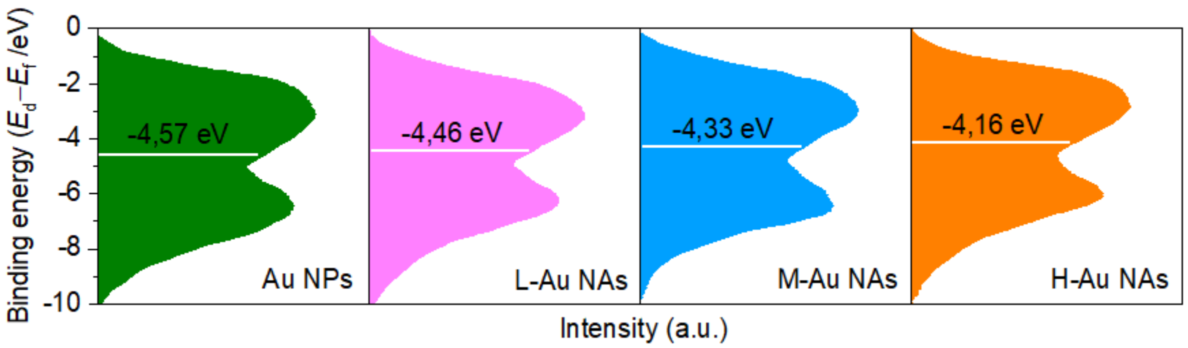

Figure S27. Surface Valence Band Photoemission Spectra measurements. Experimental surface valence band photoemission spectra of L-Au NAs, M-Au NAs and H-Au NAs.

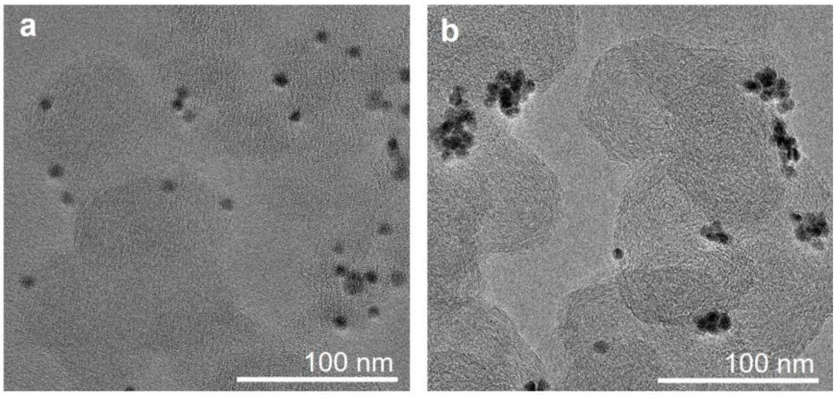

Figure S28. TEM Images of Au NPs on Carbon Support Before and After Durability Testing. TEM images of Au NPs/C before (**a**) and after (**b**) durability test. The images are intentionally defocused to show the carbon support.

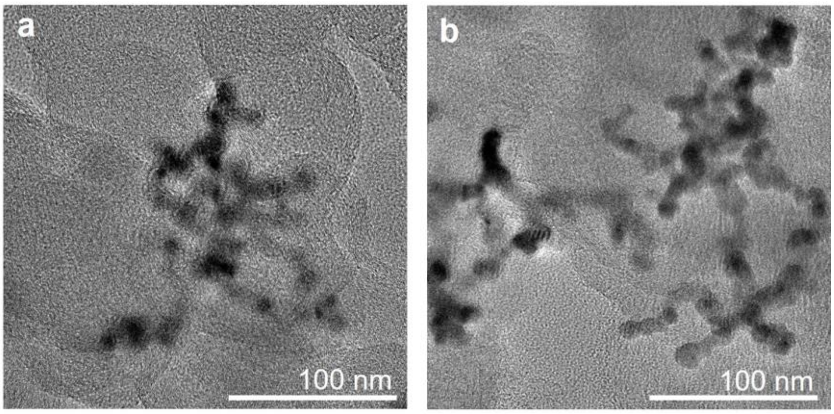

Figure S29. TEM Images of Au NAs on Carbon Support After Durability Testing. TEM images of (**a**) L-Au NAs/C and (**b**) H-Au NAs/C after durability tests. The images are intentionally defocused for showing better the carbon support.

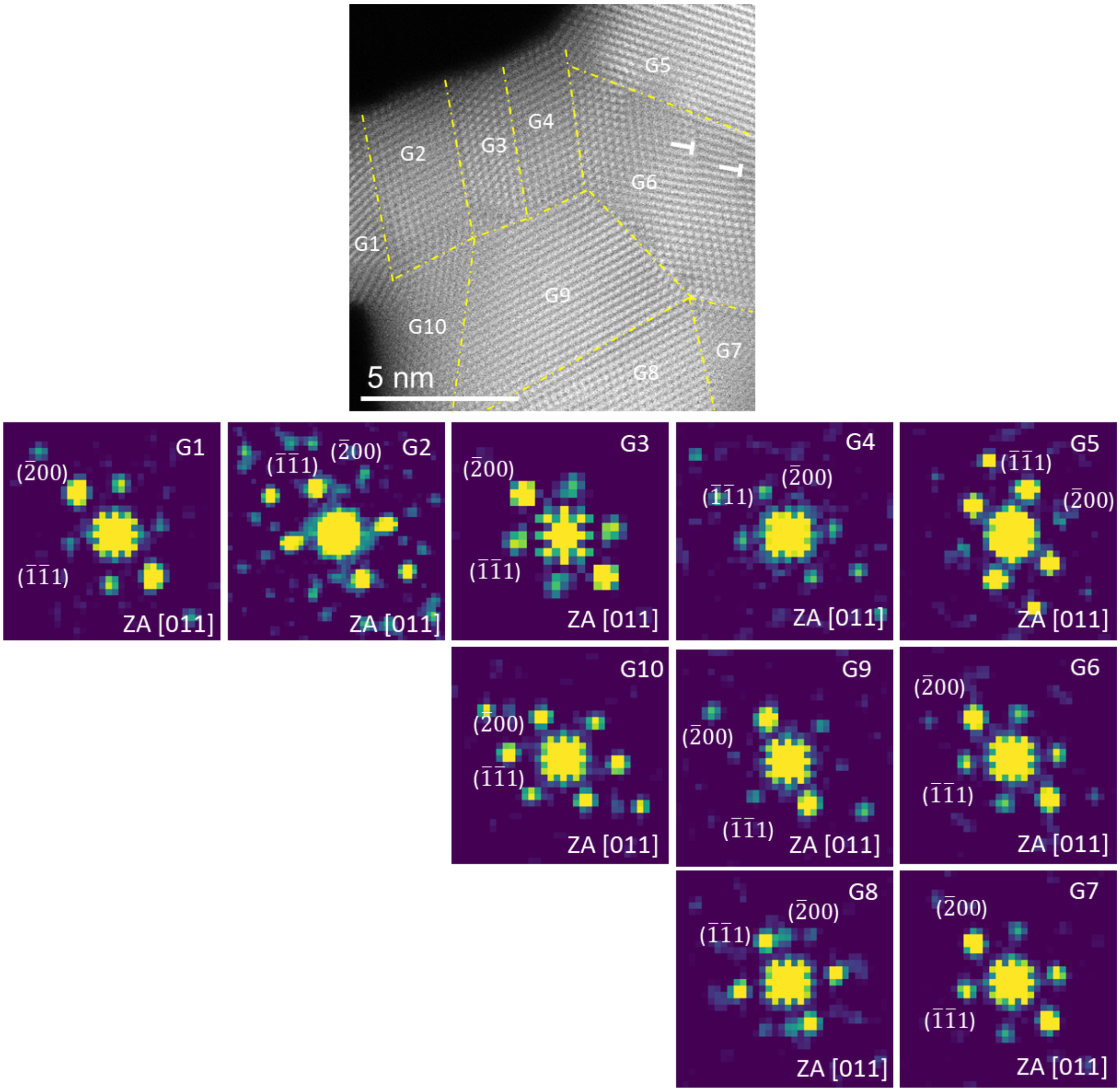

Figure S30. Identification of GB Types in H-Au NAs Post-Durability Test. HAADF image and FFT analysis for the identification of GB types based on the coincidence site lattice theory for H-Au NA after the durability test (**Figure 5d** in main text).

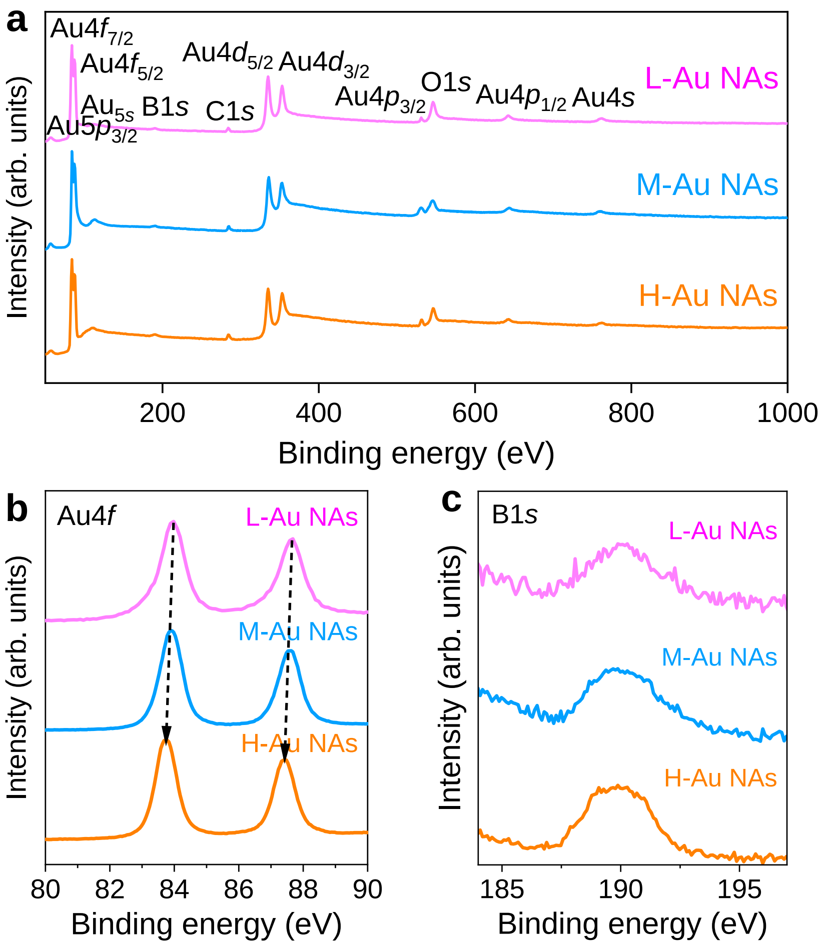

Figure S31. XPS Analysis of Au NAs. (**a**) XPS overview survey, (**b**) Au 4*f* spectra and (**c**) B 1*s* spectra of L-Au NAs, M-Au NAs and H-Au NAs.

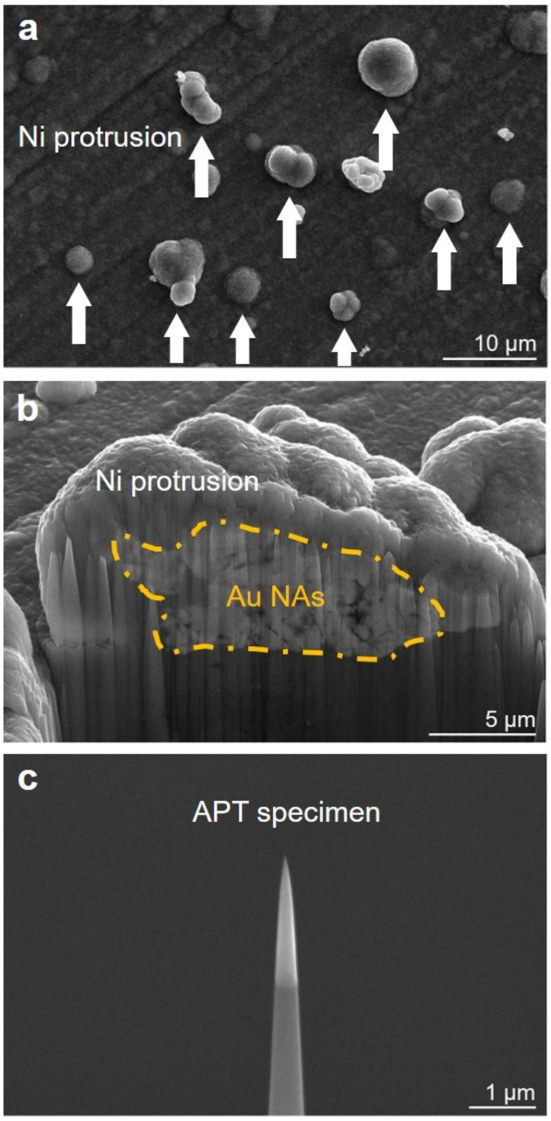

Figure S32. FIB-SEM Imaging of Au NAs Embedded in Ni Matrix. (**a**) FIB-SEM image of the protrusions (indicated by white arrows) with Au NAs embedded in Ni matrix. (**b**) The cross-sectional SEM image of one protrusion cutted by the ion beam, which indicates that Au NAs (circled by orange short dash) are embedded in the Ni matrix. (**c**) A sharpened specimen from Au NAs embedded in Ni matrix for APT measurement.

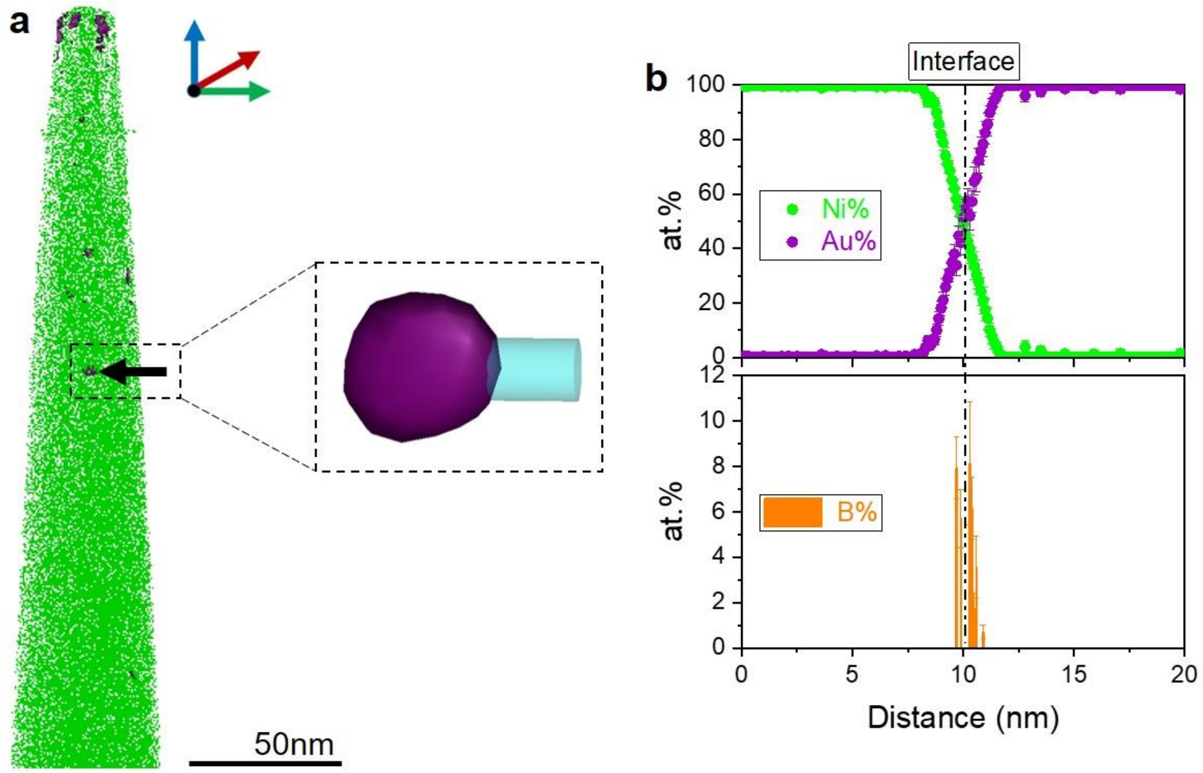

Figure S33. APT analysis of Au NPs embeded in a Ni matrix. (**a**) 3D atom map (iso-composition surface >50 at.% Au). The inset shows the NP with a cylindrical region of interest ($\Phi 5 \times 20$ nm^3, with a bin size of 0.1 nm) located perpendicular to the Ni matrix/Au NP interface. (**b**) 1D compositional profiles of the elements Ni, Au, and B contained in Au NPs shown along the direction indicated by the yellow arrow in the 3D atom map on left panel. Error bars represent the standard deviation based on a minimum of three independent measurements.

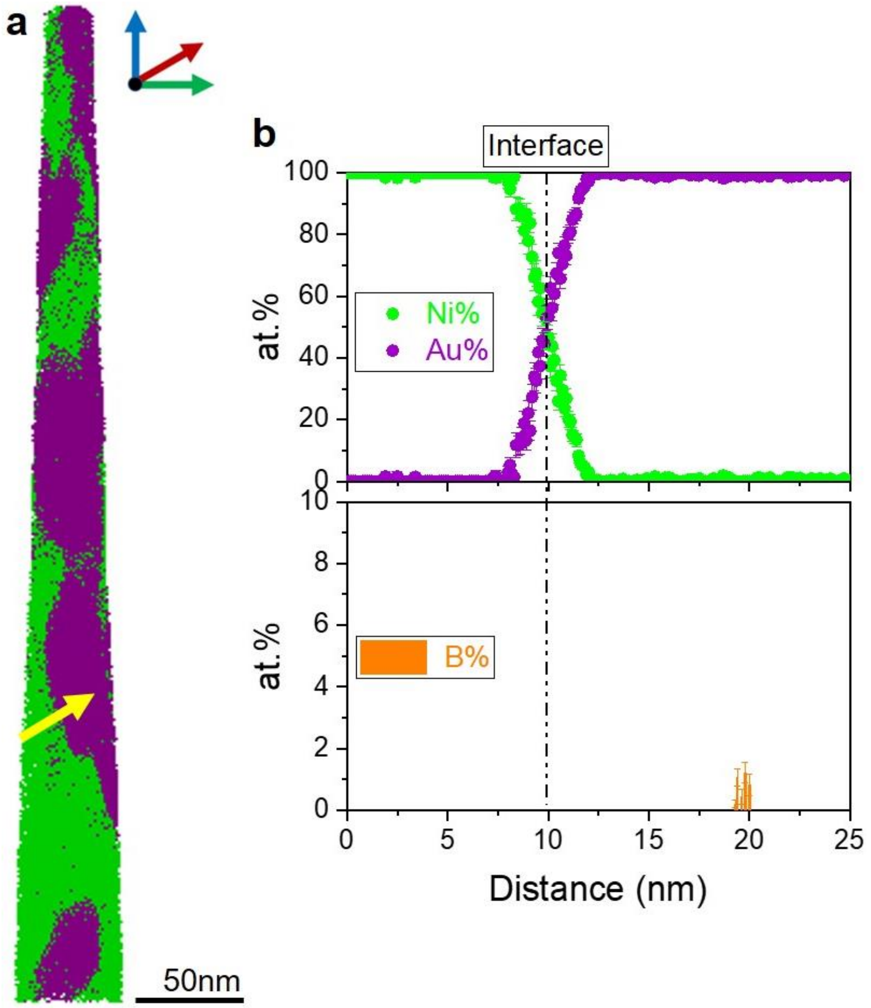

Figure S34. APT analysis of L-Au NAs embeded in a Ni matrix. (**a**) 3D atom maps of L-Au NAs fully embedded in a Ni matrix as indicated by the Au iso-surfaces (iso-composition surface >50 at.% Au) with a cylindrical region of interest (Φ5 × 25 nm^3, with a bin size of 0.1 nm) located perpendicular to the Ni matrix/L-Au NAs interface. (**b**) 1D compositional profiles of the elements Ni, Au, and B contained in L-Au NAs shown along the direction indicated by the yellow arrow in the 3D atom map on left panel. Error bars represent the standard deviation calculated from at least three independent measurements. The Gibbsian interfacial excess of B, denoted as Γ_B, is determined to be 1.1 B atoms/nm^2. It implies that a minimal quantity of B exhibits segregation at GBs within L-Au NAs.

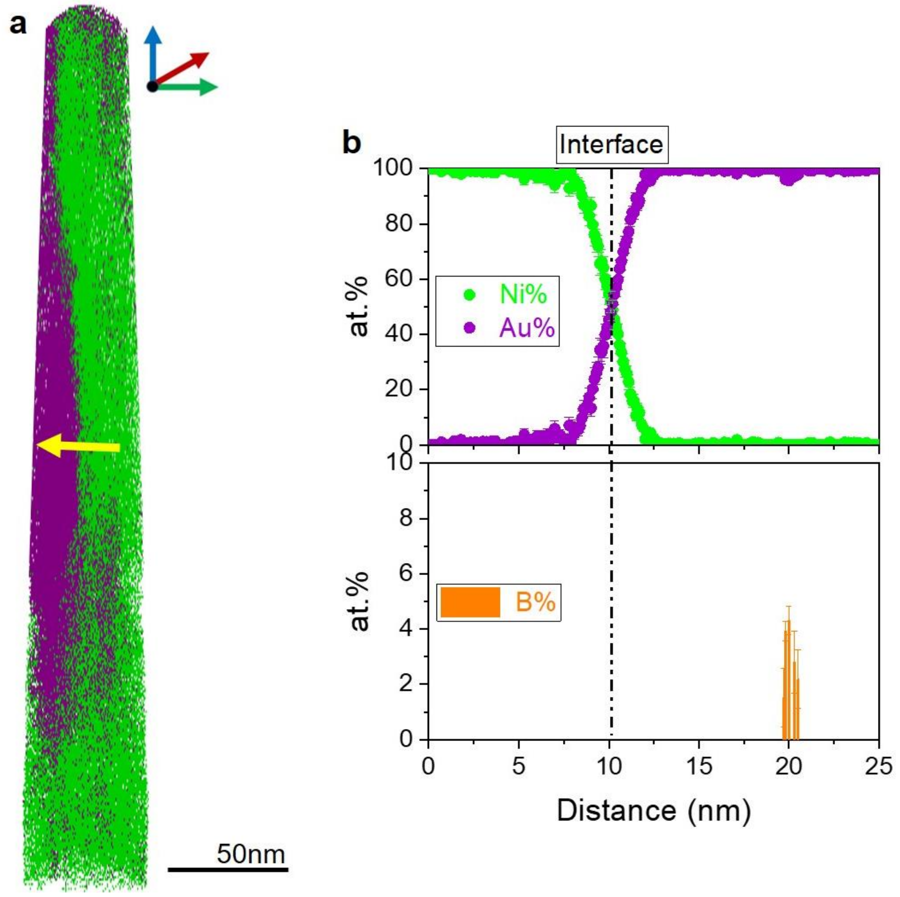

Figure S35. APT analysis of M-Au NAs embeded in a Ni matrix. APT analysis of M-Au NAs, showing the (**a**) 3D atom maps of the M-Au NAs fully embedded in a Ni matrix (Au iso-composition surface >50 at.% Au). (**b**) 1D compositional profiles of the elements Ni, Au, and B contained in M-Au NAs shown along the direction indicated by the yellow arrow in the 3D atom map on left panel (cylindrical region of interest ($\Phi 5 \times 25$ nm^3, bin size of 0.1 nm) located perpendicular to the Ni matrix/M-Au NAs interface. Error bars represent the standard deviation derived from at least three independent measurements. The determination of the Gibbsian interfacial excess of B, denoted as Γ_B, yields a value of 2.6 B atoms/nm^2. This observation indicates a greater segregation of B atoms at GBs in M-Au NAs when compared to L-Au NAs.

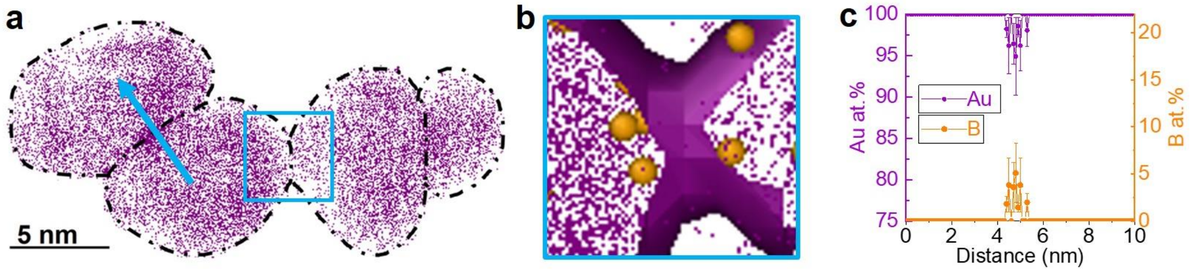

Figure S36. Tomographic Analysis of M-Au NAs. (**a**) A 2 nm thin-sliced tomogram from a 3D atom map (**Figure S35**) of M-Au NAs (iso-composition surface >90 at.% Au). (**b**) Extracted GB tomogram of M-Au NAs of the region delineated by the blue box in **Figure S36a** with the isodensity surface of 150 Au atoms/nm^3, in which the orange spheres signify B atoms. (**c**) 1D compositional profiles of identified Au and B elements of M-Au NAs along the direction indicated by the blue arrow in **Figure S36a**. Error bars indicate the standard deviation calculated from a minimum of three independent measurements.

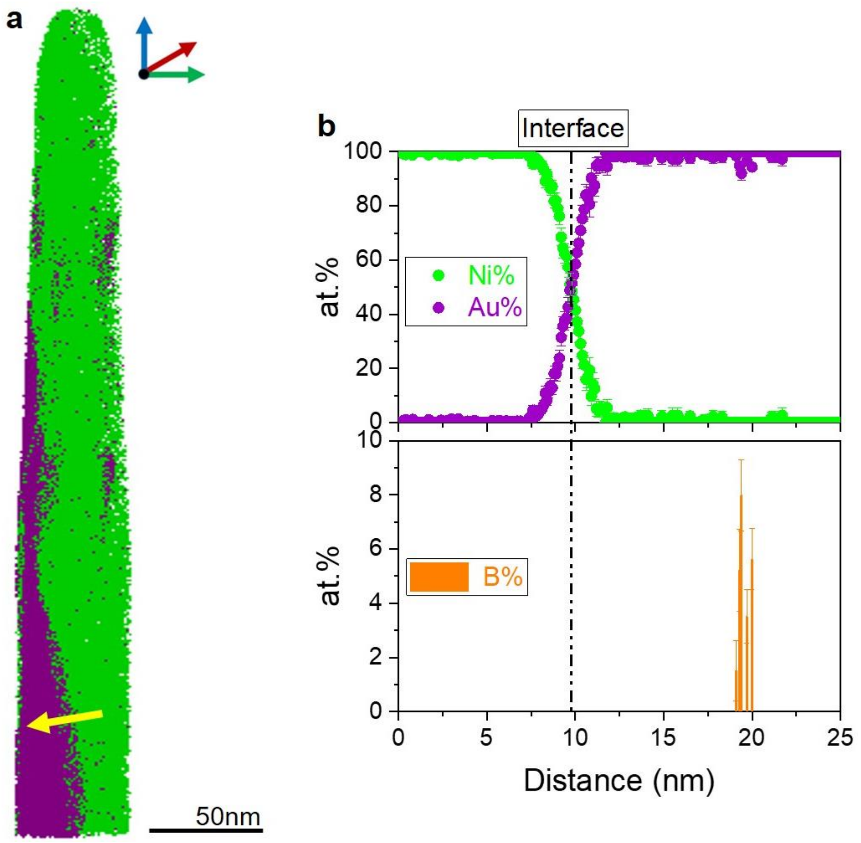

Figure S37. APT analysis of H-Au NAs embeded in a Ni matrix. (**a**) 3D atom maps of H-Au NAs embedded in a Ni matrix. (iso-composition surface >50 at.% Au) (**b**) 1D compositional profiles along the direction indicated by the yellow arrow in (a) of the elements Ni, Au, and B. Error bars represent the standard deviation derived from measurements conducted on at least three separate occasions. The computation of the Gibbsian interfacial excess of B, denoted as Γ_B, reveals a value of 4.2 B atoms/nm^2. The observed trend in the concentration of B segregated at GBs follows the order of H-Au NAs > M-Au NAs > L-Au NAs.

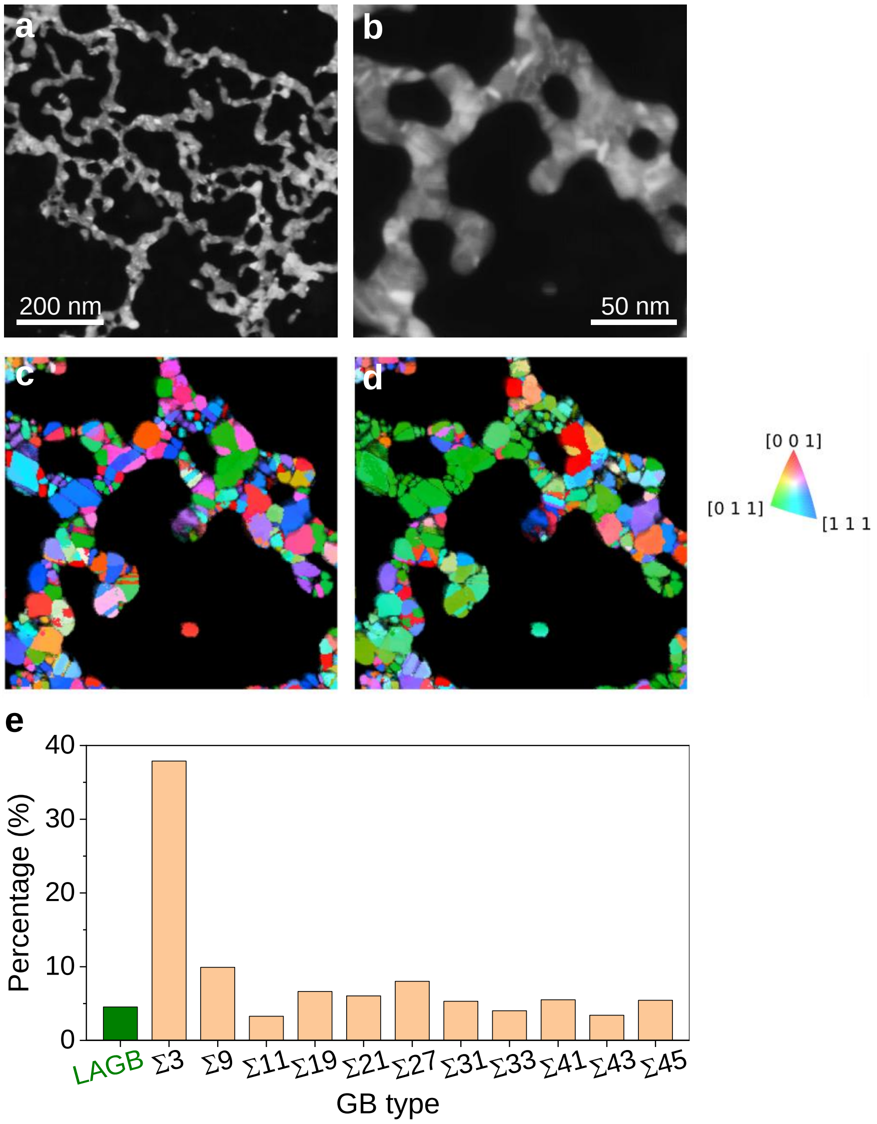

Figure S38. Characterization of B-free H-Au NAs. (**a**) Low-magnification HAADF-STEM image of B-free H-Au NAs. (**b**) HAADF-STEM image and (**c**, **d**) grain orientation maps from the corresponding 4D-STEM dataset for B-free H-Au NAs (c: in-plane orientation, d: out-of-plane orientation). (**e**) Histogram plots of the GB types derived of the analysis from over 100 grains of the 4D-STEM data.

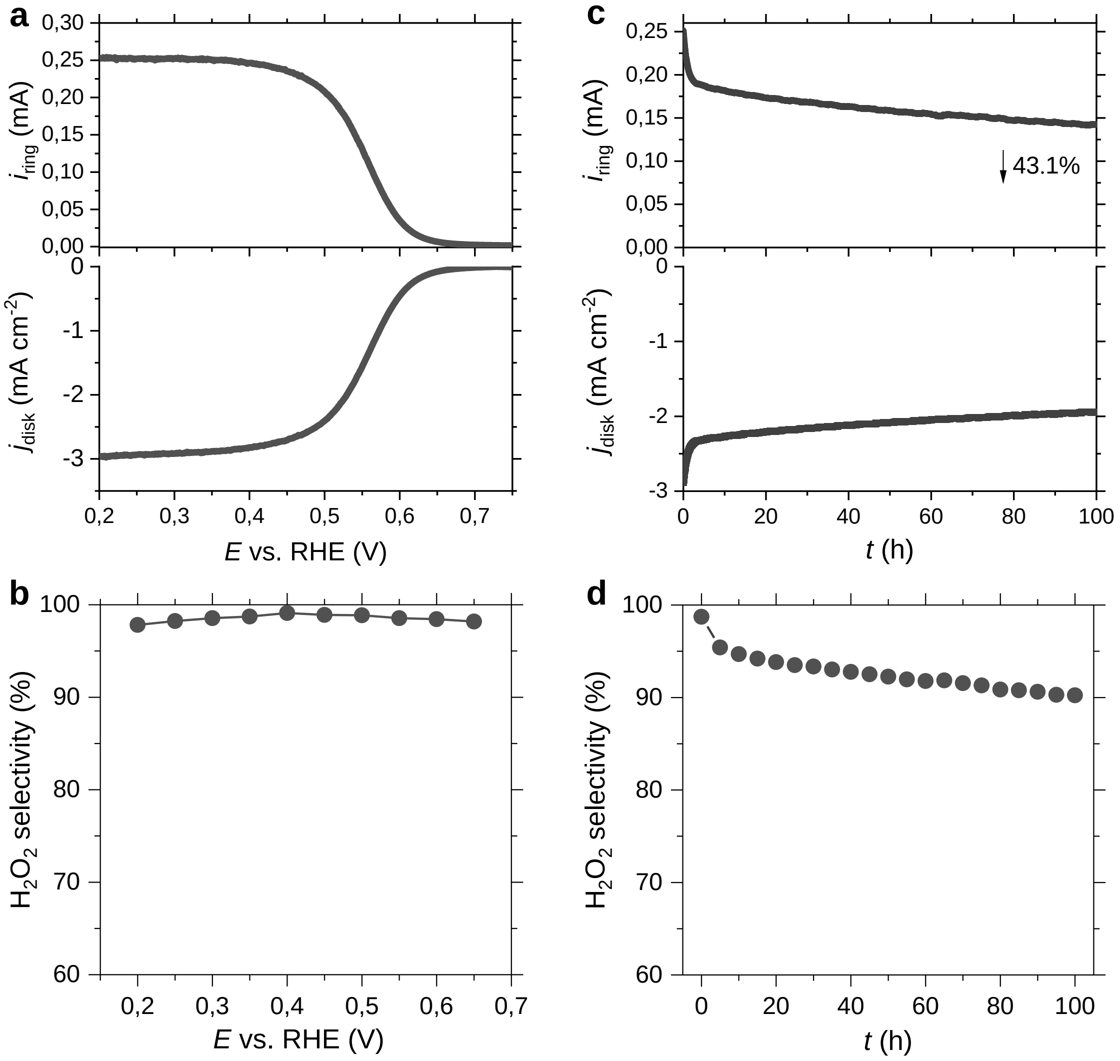

Figure S39. Electrochemical Characterization of B-free H-Au NAs. (**a**) Linear sweep voltammetry of B-free H-Au NAs recorded at 1600 rpm and a scan rate of 5 mV s^{-1} in 0.1 M $HClO_4$, together with the detected H_2O_2 currents on the ring electrode at a fixed potential of 1.2 V vs. RHE. (**b**) Comparison of the calculated H_2O_2 selectivity of B-free H-Au NAs and H-Au NAs during a potential sweep. (**c**) Durability measurements of B-free H-Au NAs at a fixed disk potential of 0.35 V. (**d**) Comparison of the calculated H_2O_2 selectivity of B-free H-Au NAs and H-Au NAs after durability tests.

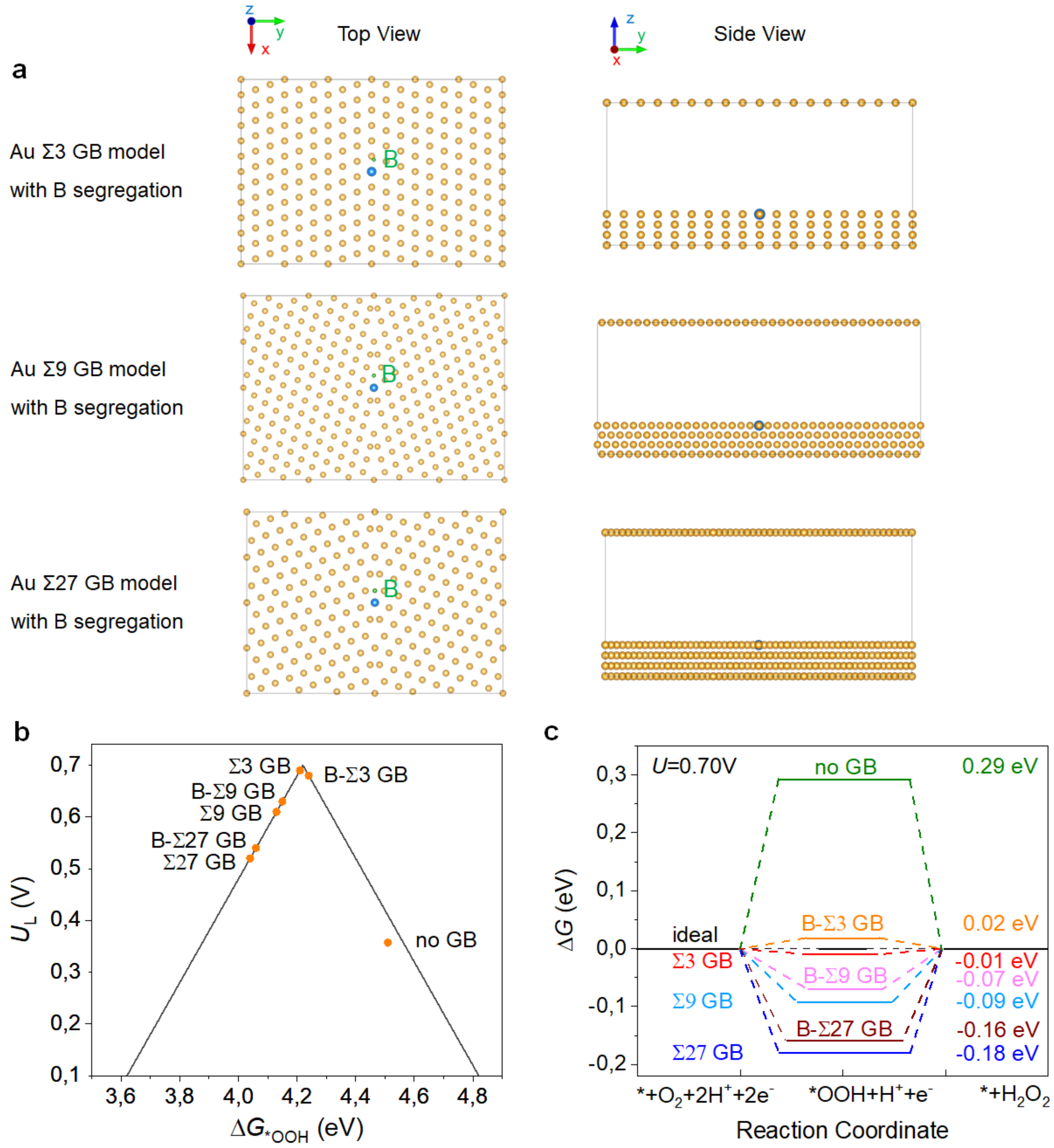

Figure S40. Modeling OOH Binding Energy on B-Segregated Au GBs. (**a**) Top and side views of the models used to calculate the OOH binding energy on B-segregated Au Σ3 GB, Σ9 GB, and Σ27 GB, with the surface Au atom for OOH binding energy calculations highlighted in deep blue and the segregated B atom shown in green. (**b**) Calculated ORR activity volcano plot illustrating the relationship between the limiting potential (U_L) and the free energy of *OOH (ΔG_{*OOH}) for the two-electron pathway to H_2O_2 on Au Σ3 GB, Σ9 GB, and Σ27 GB, both with and without B segregation. (**c**) Calculated reaction coordinate diagrams for Au Σ3 GB, Σ9 GB, and Σ27 GB, comparing scenarios with and without B segregation.

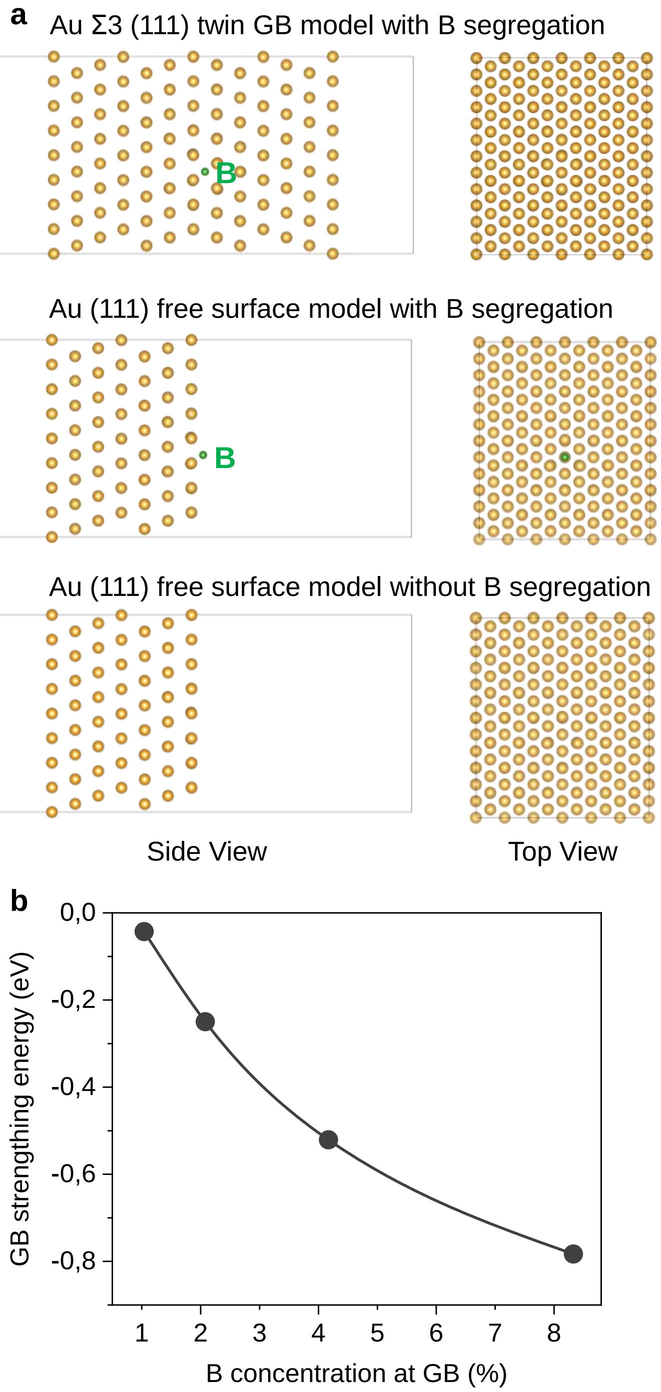

Figure S41. Modeling Strengthening Energy of B-Segregated Au Σ3 GB. (**a**) Top and side views of the models for the calculation of the strengthening energy ($E_{\text{strengthening}}$) of the B-segregated Au Σ3 GB using the following equation: $E_{\text{strengthening}} = (E_{\text{GB+B}} - E_{\text{GB}}) - (E_{\text{FS+B}} - E_{\text{FS}})$, where E_{GB} and $E_{\text{GB+B}}$ represent the total energies of the B-segregated Au Σ3 twin GB. Similarly, E_{FS} and $E_{\text{FS+B}}$ denote the total energies of the Au free surface without and with B segregation, respectively. (**b**) $E_{\text{strengthening}}$ of the B-segregated Au Σ3 GB at varying B concentrations. A negative $E_{\text{strengthening}}$ indicates that the GB is strengthened, whereas a positive $E_{\text{strengthening}}$ signifies that the GB is weakened.

Table S1. BET results of L-Au NAs, M-Au NAs, and H-Au NAs.

Sample	BET surface area (m^2/g)	Pore diameter (nm)	Pore volume (cm^3/g)
L-Au NAs	25.2	23.6	0.19
M-Au NAs	22.8	17.6	0.15
H-Au NAs	19.5	11.2	0.10

Table S2. EXAFS fitting results of Au L3 edge for Au NPs, L-Au NAs, M-Au NAs, and H-Au NAs.

Sample	CN	R (Å)	σ^2 (10^{-3} Å^2)	ΔE_0 (eV)	R factor
Au NPs	11.8±0.2	2.62±0.02	2.9±1.4	4.4±1.2	0.013
L-Au NAs	10.9±0.3	2.67±0.01	2.1±1.1	2.3±1.5	0.008
M-Au NAs	9.9±0.2	2.73±0.02	3.6±1.5	4.1±2.0	0.017
H-Au NAs	8.7±0.2	2.78±0.02	2.8±1.2	3.5±1.7	0.012

CN: coordination number; R: bond length; σ: Debye-Waller factor; ΔE_0: inner potential shift; R factor: goodness of the fit.

Table S3. EXAFS fitting results of Au L3 edge for Au NPs and H-Au NAs after ORR tests.

Sample	CN	R (Å)	σ^2 (10^{-3} Å^2)	ΔE_0 (eV)	R factor
Au NPs after ORR	12.0±0.1	2.62±0.03	2.5±1.2	4.1±1.7	0.011
H-Au NAs after ORR	8.7±0.2	2.78±0.03	3.1±1.3	3.7±1.3	0.013

CN: coordination number; R: bond length; σ: Debye-Waller factor; ΔE_0: inner potential shift; R factor: goodness of the fit.

Table S4. Comparative evaluation of two-electron oxygen reduction reaction (ORR) performance in 0.1 M $HClO_4$ media between Au catalysts synthesized in the present study and state-of-the-art two-electron ORR nanocatalysts reported in recent publications.

Catalyst	E_{onset} vs. RHE	Selectivity at 0.6 V	Selectivity at 0.5 V	Selectivity at 0.4 V	Selectivity at 0.3 V	Selectivity at 0.2 V	Ref.
Pt–Hg NPs	0.54 V	77%	93%	95%	91%	83%	Nat. Mater. 12, 1137–1143 (2013)
PtP_2 NCs	0.66 V	73%	80%	87%	99%	97%	Nat. Commun. 11, 3928 (2020)
partially oxidized Pd with defect carbon	0.60 V	–	–	92%	91%	88%	Nat. Commun. 11, 2178 (2020)
Pt/HSC	0.51 V	93%	94%	95%	94%	94%	Nat. Commun. 7, 10922 (2016)
h-Pt_1-CuSx NPs	0.61 V	95%	95%	96%	95%	93%	Chem 5, 2099–2110 (2019)
N-doped single-wall carbon nanohorns	0.4 V	–	–	–	97%	97%	Chem 4, 106–123 (2018)
0.35% Pt/TiN	0.63 V	38%	45%	52%	55%	57%	Angew. Chem. Int. Ed. 55, 2058 (2016)
Hierarchically porous carbon	0.2 V	–	–	–	–	96%	Angew. Chem. Int. Ed. 54, 6837–6841 (2015)
Pd-Hg NPs	0.62 V	91%	99%	97%	92%	85%	Nano Lett. 14, 1603 (2014)
$Au_{0.92}Pd_{0.08}$ NPs	0.55 V	–	90%	92%	92%	87%	J. Am. Chem. Soc. 133, 19432 (2011)
Au–Pd_2Hg_5 aerogel	0.49 V	–	–	96%	92%	92%	Adv. Mater. 35, 2211512 (2023)
Pd_4Se NPs	0.39 V	–	90%	92%	92%	90%	Nano Energy 89, 106480 (2021)
**Au NPs**	**0.5 V**	**–**	**77.9%**	**69.9%**	**68.9%**	**68.8%**	**This work**
**L-Au NAs**	**0.57 V**	**–**	**80.0%**	**79.7%**	**78.9%**	**79.6%**	**This work**
**M-Au NAs**	**0.61 V**	**90.7%**	**90.8%**	**90.5%**	**90.1%**	**91.5%**	**This work**
**H-Au NAs**	**0.66 V**	**99.5%**	**99.0%**	**98.9%**	**98.7%**	**98.0%**	**This work**

Table S5. EXAFS fitting results of Au L3 edge for B-free H-Au NAs before and after ORR tests.

Sample	CN	R (Å)	σ^2 (10^{-3} Å^2)	ΔE_0 (eV)	R factor
B-free H-Au NAs before ORR	8.8±0.3	2.76±0.02	3.4±1.6	4.2±1.5	0.019
B-free H-Au NAs after ORR	9.6±0.2	2.70±0.03	3.2±1.4	3.9±1.2	0.015

CN: coordination number; R: bond length; σ: Debye-Waller factor; ΔE_0: inner potential shift; R factor: goodness of the fit.

Supplementary References

1. De Backer, A.; Van den Bos, K.; Van den Broek, W.; Sijbers, J.; Van Aert, S., StatSTEM: An efficient approach for accurate and precise model-based quantification of atomic resolution electron microscopy images. *Ultramicroscopy* **2016,** *171*, 104-116.
2. Peters, J. J.; Beanland, R.; Alexe, M.; Cockburn, J. W.; Revin, D. G.; Zhang, S. Y.; Sanchez, A. M., Artefacts in geometric phase analysis of compound materials. *Ultramicroscopy* **2015,** *157*, 91-97.
3. Ophus, C.; Zeltmann, S. E.; Bruefach, A.; Rakowski, A.; Savitzky, B. H.; Minor, A. M.; Scott, M. C., Automated crystal orientation mapping in py4DSTEM using sparse correlation matching. *Microscopy and microanalysis* **2022,** *28* (2), 390-403.
4. Geng, X.; Li, S.; Heo, J.; Peng, Y.; Hu, W.; Liu, Y.; Huang, J.; Ren, Y.; Li, D.; Zhang, L., Grain-Boundary-Rich Noble Metal Nanoparticle Assemblies: Synthesis, Characterization, and Reactivity. *Advanced Functional Materials* **2022,** *32* (34), 2204169.
5. Grabow, L. C.; Gokhale, A. A.; Evans, S. T.; Dumesic, J. A.; Mavrikakis, M., Mechanism of the water gas shift reaction on Pt: First principles, experiments, and microkinetic modeling. *The Journal of Physical Chemistry C* **2008,** *112* (12), 4608-4617.
6. Hohenberg, P.; Kohn, W., Inhomogeneous electron gas. *Physical review* **1964,** *136* (3B), B864.
7. Kohn, W.; Sham, L. J., Self-consistent equations including exchange and correlation effects. *Physical review* **1965,** *140* (4A), A1133.
8. Kresse, G.; Furthmüller, J., Efficiency of ab-initio total energy calculations for metals and semiconductors using a plane-wave basis set. *Computational materials science* **1996,** *6* (1), 15-50.
9. Kresse, G.; Furthmüller, J., Efficient iterative schemes for ab initio total-energy calculations using a plane-wave basis set. *Physical review B* **1996,** *54* (16), 11169.
10. Larsen, A. H.; Mortensen, J. J.; Blomqvist, J.; Castelli, I. E.; Christensen, R.; Dułak, M.; Friis, J.; Groves, M. N.; Hammer, B.; Hargus, C., The atomic simulation environment—a Python library for working with atoms. *Journal of Physics: Condensed Matter* **2017,** *29* (27), 273002.
11. Phatak, A. A.; Delgass, W. N.; Ribeiro, F. H.; Schneider, W. F., Density functional theory comparison of water dissociation steps on Cu, Au, Ni, Pd, and Pt. *The Journal of Physical chemistry c* **2009,** *113* (17), 7269-7276.
12. Perdew, J. P.; Burke, K.; Ernzerhof, M., Generalized gradient approximation made simple. *Physical review letters* **1996,** *77* (18), 3865.
13. Hammer, B.; Hansen, L. B.; Nørskov, J. K., Improved adsorption energetics within density-functional theory using revised Perdew-Burke-Ernzerhof functionals. *Physical review B* **1999,** *59* (11), 7413.
14. Blöchl, P. E., Projector augmented-wave method. *Physical review B* **1994,** *50* (24), 17953.
15. Kresse, G.; Joubert, D., From ultrasoft pseudopotentials to the projector augmented-wave method. *Physical review b* **1999,** *59* (3), 1758.
16. Monkhorst, H. J.; Pack, J. D., Special points for Brillouin-zone integrations. *Physical review B* **1976,** *13* (12), 5188.
17. Kulkarni, A.; Siahrostami, S.; Patel, A.; Nørskov, J. K., Understanding catalytic activity trends in the oxygen reduction reaction. *Chemical Reviews* **2018,** *118* (5), 2302-2312.
